# Engineering superconducting qubits to reduce quasiparticles and charge noise

**DOI:** 10.1038/s41467-022-34727-2

**Published:** 2022-11-23

**Authors:** Xianchuang Pan, Yuxuan Zhou, Haolan Yuan, Lifu Nie, Weiwei Wei, Libo Zhang, Jian Li, Song Liu, Zhi Hao Jiang, Gianluigi Catelani, Ling Hu, Fei Yan, Dapeng Yu

**Affiliations:** 1 Shenzhen Institute for Quantum Science and Engineering, Southern University of Science and Technology, Shenzhen, Guangdong China; 2 International Quantum Academy, Shenzhen, Guangdong China; 3 Guangdong Provincial Key Laboratory of Quantum Science and Engineering, Southern University of Science and Technology, Shenzhen, Guangdong China; 4 Department of Physics, Southern University of Science and Technology, Shenzhen, Guangdong China; 5 State Key Laboratory of Millimeter Waves, School of Information Science and Engineering, Southeast University, Nanjing, China; 6 JARA Institute for Quantum Information (PGI-11), Forschungszentrum Jülich, 52425 Jülich, Germany; 7 Quantum Research Centre, Technology Innovation Institute, Abu Dhabi, UAE

**Keywords:** Qubits, Electronic properties and materials

## Abstract

Identifying, quantifying, and suppressing decoherence mechanisms in qubits are important steps towards the goal of engineering a quantum computer or simulator. Superconducting circuits offer flexibility in qubit design; however, their performance is adversely affected by quasiparticles (broken Cooper pairs). Developing a quasiparticle mitigation strategy compatible with scalable, high-coherence devices is therefore highly desirable. Here we experimentally demonstrate how to control quasiparticle generation by downsizing the qubit, capping it with a metallic cover, and equipping it with suitable quasiparticle traps. Using a flip-chip design, we shape the electromagnetic environment of the qubit above the superconducting gap, inhibiting quasiparticle poisoning. Our findings support the hypothesis that quasiparticle generation is dominated by the breaking of Cooper pairs at the junction, as a result of photon absorption by the antenna-like qubit structure. We achieve record low charge-parity switching rate (<1 Hz). Our aluminium devices also display improved stability with respect to discrete charging events.

## Abstract (web summary)

Quasiparticles, or broken Cooper pairs, are a major source of decoherence in superconducting qubits but their origin is debated. Pan et al. confirm the dominant mechanism due to photon absorption in the Josephson junction and demonstrate mitigation strategies based on tuning of the qubit geometry.

## Introduction

Quantum computers and simulators are highly anticipated transformative technologies, and superconducting quantum circuits based on Josephson junctions are a leading candidate for their realization. The proper functioning of superconducting circuits requires a pristine environment to protect the collective behaviour of Cooper pairs. An acknowledged potential danger is quasiparticle poisoning, that is, the presence of broken pairs ubiquitously seen in superconducting devices; these quasiparticles can be a significant source of decoherence in qubits based on Josephson junctions^1,2^. Moreover, recent studies with superconducting circuits^3–6^ have shown that energy deposited in the substrate may cause quasiparticle generation not only locally (i.e., in a single qubit) but also across multiple qubits within a short time, leading to correlated errors that can impede quantum error correction. Therefore, a deeper understanding of the generation mechanisms of quasiparticles, from ionizing radiation^7^ to stray photons^8^, is imperative.

Quasiparticles have been intensely investigated over the last decade^9–12^. Puzzlingly, experiments conducted using various devices unanimously suggest a much higher number of quasiparticles at experimental temperatures (typically ~10 mK) than expected in thermal equilibrium, a phenomenon that is not yet fully understood. The number of quasiparticles is determined by the balance between generation, i.e., the breaking of a Cooper pair into two quasiparticles, and recombination, the reverse process. While recombination is determined by material properties that can be difficult to modify, generation can be controlled to some extent, for example, by using phonon traps^13^. For small superconducting islands, schemes to pump out quasiparticles have been developed^14^ and protection by a ground plane has been shown to reduce quasiparticle generation^15^. Alternatively, trapping quasiparticles in normal-metal islands, so that they cannot tunnel through Josephson junctions, can also protect qubits^16^. However, a mitigation method that is compatible with state-of-the-art quantum processor designs^17^ has not yet been demonstrated.

Here we experimentally investigate how variations in our superconducting qubit design affect the rate of quasiparticle generation and show that the main quasiparticle source is local: quasiparticles originate from the breaking of Cooper pairs at the Josephson junction via the absorption of stray photons. This corroborates the conjecture that photons with energy greater than twice the superconducting energy gap and whose absorption is mediated by the antenna-like structure of the qubit are responsible for the observed excess quasiparticles^8,18,19^. Leveraging the design flexibility of superconducting circuits, particularly flip-chip technology, we demonstrate convenient control of the antenna mode and hence the quasiparticle generation, achieving an exceedingly low charge-parity switching rate (Γ_*P*_ ≲ 1 Hz) in our aluminium qubits. The charge offset stability is also improved, and the occurrence rate of strong charge jumps (jump amplitudes >0.1*e*, where *e* is an electron charge) is on the order of 0.01 mHz. In addition, the measured temperature dependence of the charge-parity switching rates is consistent with quasiparticles being thermally excited out of the capacitor pads, which act as superconducting traps^20^, and into the junction leads.

## Results

### Devices and quasiparticle generation mechanism

**Fig. 1:**
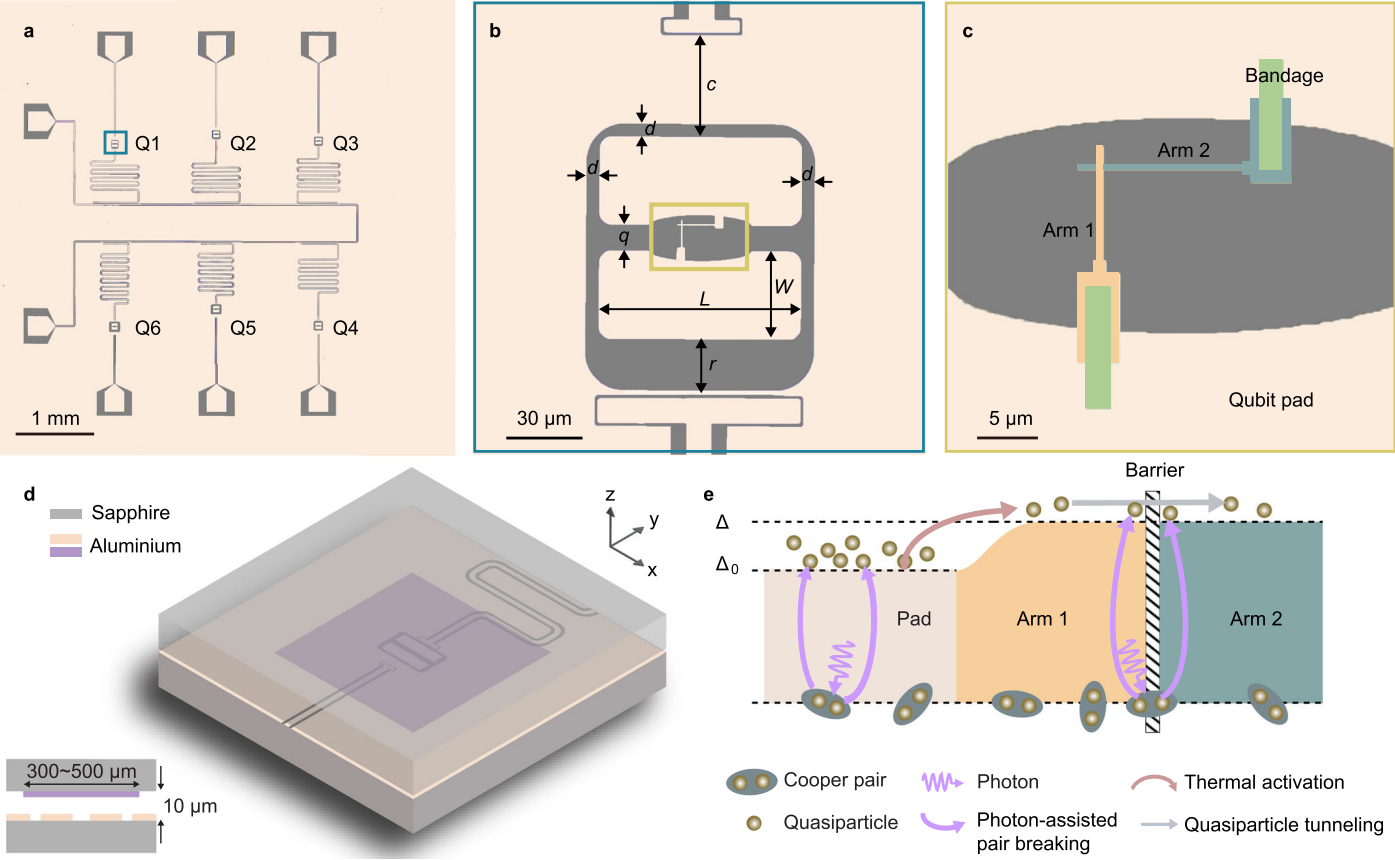
Device layout and photon-assisted quasiparticle generation. **a** Optical micrograph of a planar-design 6 mm × 6 mm sample chip. The light area indicates the base aluminium layer; the dark area indicates the exposed sapphire substrate. Each qubit (Q1--Q6) has a dedicated charge drive line (straight transmission line) and a dedicated readout resonator (meandering transmission line). The six resonators share a common feed line for a multiplexed readout. **b** Close-up view of Q1 (blue rectangle in **a**), showing two aluminium pads (length *L* and width *W*) floating inside an aperture. The pad-to-resonator distance is *r*; the pad-to-ground distance on the other three sides is *d*; the pad-to-drive-line distance is *c*; the pad-to-pad distance is *q*. In the shown case, *L* = 80 μm, *W* = 35 μm, *r* = 23 μm, *d* = 5 μm, *c* = 41 μm, *q* = 10 μm. The qubits shown in **a** have varying pad-to-ground distances (*d* = 5 − 30 μm). **c** Close-up view of the junction area (yellow rectangle in **b**). Two layers (colour-coded) of aluminium film are deposited on the base layer to form the junction, i.e., the region where the two thin strips (Arm1 and Arm2) overlap. An additional aluminium layer (10 μm × 2 μm rectangular patches) forms the bandage structures that are used to improve the galvanic contact^46^. The film thicknesses are 100 nm for the pads, 30 nm for Arm1, 40 nm for Arm2, and 200 nm for the bandages. **d** Schematic of a vertically integrated device with the qubit, resonator and drive line patterned on the bottom die and a square-shaped aluminium pad (purple) on the top die floating above the qubit. The inset on the bottom left shows a cross-sectional view of the device. **e** Schematic illustrating the quasiparticle processes. Δ_0_ and Δ are the superconducting energy gaps of the pads and the junction leads, respectively.

Our devices have two different types of architectures, planar and vertically integrated^21–23^, both consisting of aluminium on c-plane sapphire substrates. A planar sample consists of six qubits separated by at least 1.3 mm (Fig. 1a), with each qubit (transition frequency between the ground and excited state *ω*_ge_) coupled to a local resonator (frequency *ω*_r_) for dispersive readout^24^ and to a dedicated control line (feeding both direct current and radio frequency signals). As shown in Fig. 1b, the qubits share a floating transmon design^25^, two rectangular-shaped capacitor pads (charging energy *E*_C_ = *e*^2^/2*C*_Σ_, where *C*_Σ_ is the total capacitance) shunting a Josephson junction (Josephson energy *E*_J_). We fabricated Manhattan-style Josephson junctions using two aluminium leads, Arm1 and Arm2 in Fig. 1c, that extend from the pads and overlap each other, separated by an aluminium oxide barrier. In addition, we fabricated a second type of device using flip-chip technology to cover the qubit structure with a floating aluminium cap separated by 10 μm (Fig. 1d). In both the capped and uncapped devices, we added variations in the circuit design across the different qubits to investigate the generation mechanism of the nonequilibrium quasiparticles. We explored an extended parameter regime of the *E*_J_/*E*_C_ ratio (2–30) between the transmon and the Cooper pair box or charge qubit^26–28^, which retains sensitivity to charge fluctuations and quasiparticle tunnelling. The samples were packaged in aluminium and copper boxes that were thermally anchored to the mixing chamber stage (<10 mK) of a dilution refrigerator. Similar to other studies^18,29,30^, we find that careful shielding and filtering are important to reduce quasiparticles. See Supplementary Note 1 ~ 2 (Supplementary Material) for more information concerning the device and experimental setup.

The aluminium film is thinner at the junction leads (30–40 nm) than at the pads (100 nm); therefore, the superconducting gap frequency near the junction (f^*^ = 2Δ/*h* ≈ 2 × 217 μeV/*h* = 105 GHz) is higher than that in the pads (*f*_0_ = 2Δ_0_/*h* ≈ 2 × 180 μeV/*h* = 87 GHz)^31,32^. Accordingly, we hypothesize the following scenario for the generation and tunnelling processes of nonequilibrium quasiparticles (Fig. 1e). Photons or phonons with energy greater than twice the superconducting energy gap can break a Cooper pair and create two quasiparticles. Such bulk generation is more likely in the pads than in the arms, because of the much larger area and volume of the pads. However, these nonequilibrium quasiparticles may not directly contribute to tunnelling across the junction, unless, for example, they are excited by phonons to overcome the gap difference between the thinner junction arms and the pads. Conversely, the coherent tunnelling of a Cooper pair across the junction can accompany a photon absorption event, as a form of photon-assisted tunnelling^33^ that breaks the pair and creates one quasiparticle on each side of the barrier. Therefore, by measuring the quasiparticle tunnelling rate, one can infer the absorption efficiency of sub-terahertz (~100 GHz) photons.

The qubit Hamiltonian can be expressed in the form of a generalized Cooper-pair box^18^:

1 $${\hat{H}}_{{{{{{{{\rm{q}}}}}}}}}=4{E}_{{{{{{{{\rm{C}}}}}}}}}{\left(\hat{n}-{n}_{{{{{{{{\rm{g}}}}}}}}}+\frac{P-1}{4}\right)}^{2}-{E}_{{{{{{{{\rm{J}}}}}}}}}\cos \hat{\phi },$$

where $\hat{n}$ is the number of Cooper pairs that have traversed the junction and $\hat{\phi }$ is the superconducting phase difference across the junction. *n*_g_ indicates the offset charge in units of 2*e* and the Hamiltonian has a 2*e*-periodicity. *P* is the charge parity of the circuit, where *P* = 1 corresponds to even parity and *P* = − 1 corresponds to odd parity. The Hamiltonian implies that a change in the charge parity of the junction electrodes is equivalent to a shift of 1*e* in the offset charge. Compared to the usual transmon Hamiltonian^25^, in which the parity is conventionally fixed to be even, the system described by Eq. (1) has twice as many eigenstates, one for each parity.

### Measuring the charge-parity switch rate

**Fig. 2:**
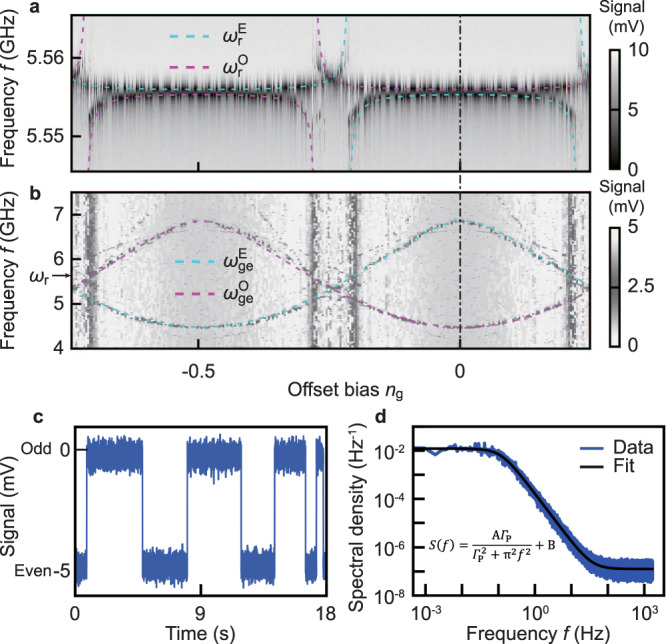
Spectroscopy and charge-parity detection. **a** Resonator (*ω*_r_) and **b** qubit spectroscopy as a function of the offset charge bias *n*_g_, showing 2*e* periodicity and a shift of 1*e* between even and odd parity. The dashed lines are the identified resonator frequency ${\omega }_{{{\rm{r}}}}^{{{{{{{{\rm{E(O)}}}}}}}}}$ and the g-e transition frequency of the qubit ${\omega }_{{{\rm{ge}}}}^{{{{{{{{\rm{E(O)}}}}}}}}}$ (the superscript letter E and O indicate even and odd parity, respectively) from fitting to the Jaynes-Cumming model. The resonator spectrum was acquired by a network analyzer at a rate of 0.2 s per offset bias or vertical linecut, while the qubit spectrum was obtained from pulsed measurements with each data point taking ~0.1 s. See Supplementary Note 3 (Supplementary Material) for the other identified transitions. **c** Example of the time evolution (time interval: 0.3 ms; total length 18 s) of the charge parity measured at *n*_g_ = 0 showing random telegraph behaviour between even (*P* = 1) and odd (*P* = − 1) parity. **d** Power spectrum of the charge-parity fluctuations obtained from 1200 repetitions of the measurement in **c**. The inset shows the Lorentzian fitting function, where Γ_*P*_ (the average switching rate), A and B are fitting parameters. In the illustrated case, the extracted charge-parity lifetime *T*_*P*_ = 1/Γ_*P*_ = 2.7s. The white noise (offset term *B*) is due to the sampling noise.

Both the single-tone resonator and two-tone qubit spectroscopy (Fig. 2a and b, respectively) exhibit random switching between the two spectral curves corresponding to the different parities. In the displayed case, the qubit is in the charge regime (*E*_J_/*E*_C_ ~ 3), leading to distinct qubit transition frequencies for the two parities, ${\omega }_{{{{{{{{\rm{ge}}}}}}}}}^{{{{{{{{\rm{E}}}}}}}}}/2\pi=6.850$ GHz and ${\omega }_{{{{{{{{\rm{ge}}}}}}}}}^{{{{{{{{\rm{O}}}}}}}}}/2\pi=4.478$ GHz at *n*_g_ = 0, and hence different dispersive shifts of the resonator frequency. By fitting both spectra to Eq. (1) and the Jaynes-Cummings model^24^, we can extract the actual device parameters: *E*_J_/*h* = 4.6 GHz, *E*_C_/*h* = 1.4 GHz, and the qubit-resonator coupling *g*/*h* = 24 MHz.

To track how the charge parity evolves over time, we repeatedly measured the transmitted signal. With our approach, events faster than the sampling rate of 3.3 kHz may be missed. However, if the parity switching is a random process without strong correlation, the only missed events would be those with parity consecutively switching for an even number of times during a short time interval. Such events are not the same type of random telegraph processes studied in this work. Indeed, a typical trace is shown in Fig. 2c, displaying a telegraph signal that randomly switches between even and odd parity every few seconds. We acquired a few hundred such traces and computed their power spectral density (Fig. 2d); the Lorentzian spectral shape is consistent with a random telegraph process. The spectral width is proportional to the average switching rate Γ_*P*_; in this case, *T*_*P*_ = 1/Γ_*P*_ = 2.7s, which is a state-of-the-art result for superconducting qubits. Previously reported *T*_*P*_ values range from 1 ms^34^ to ~10 ms under similar shielding and filtering conditions^18^, and have recently been prolonged to 100 ms level by creating a light-tight environment^30^. Our result—more than one order of magnitude better without sophisticated shielding—implies the significant influence of the device geometry. This influence, as we discuss next, is much stronger than the variation in switching rates (about a factor of 2) between devices with identical design measured in the same cooldown, and between different cooldowns for a given device (see Supplementary Fig. 5 (Supplementary Material) for details).

### Effect of circuit geometry on parity switching

**Fig. 3:**
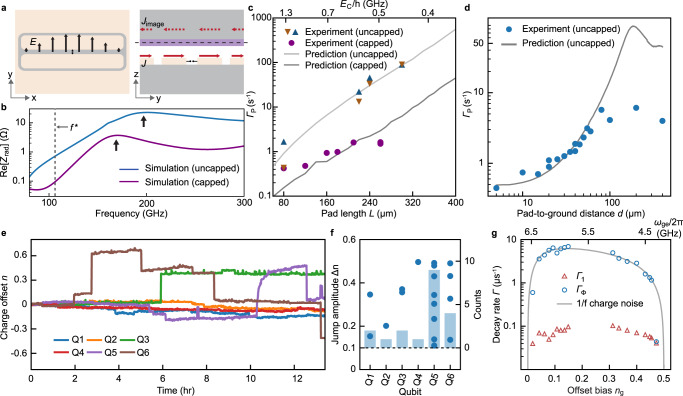
Effect of circuit geometry on parity switching, charge offset stability, and coherence. **a** Top view of the electric field (*E*, arrows) of the fundamental radiation mode formed by the floating transmon structure (left). Mode current (*J*, solid arrows) and its image (*J*_image_, dashed arrows) on the two sides of the aluminium cap indicated by the dashed line (right). **b** Real part of the simulated input impedance *Z*_rad_ of a typical qubit (*L* = 260 μm, *W* = 35 μm) with and without a cap. The arrows indicate the peaks corresponding to the fundamental mode. The dashed line indicates the superconducting gap frequency at 105 GHz. **c** Measured parity switching rates for capped (dots) and uncapped (triangles) qubits with varying pad size plotted as a function of the pad length. In the uncapped case, the pad width is also varying, but the effect is much smaller. The solid lines indicate finite-element simulation predictions. The top axis indicates the charging energy *E*_C_ that corresponds to the different pad lengths in the capped case. **d** Measured (dots) and simulated (line) parity switching rate of qubits with varying pad-to-ground distance *d*. **e** Offset charge drift for six qubits on a single chip (Fig. 1a) with the same pad size but varying pad-to-ground distance (*d* = 5 − 30 μm), simultaneously monitored over a 13-h period. **f** Amplitudes (dots, left axis) and total counts (bars, right axis) of all offset charge jumps (∣Δ*q*∣) >0.1e identified in the data in **c** and the extended data (Supplementary Material) during a total of 40 h of monitoring. **g** Measured relaxation (Γ_1_) and pure-dephasing (Γ_ϕ_) rates of Q1 as a function of *n*_g_ sampled over half a period. The top axis indicates the corresponding qubit frequency. The solid line is fit to the 1/*f* charge noise model. It is difficult to characterize the qubit around *n*_g_ = 0.25 because of the stronger sensitivity to charge noise around this bias and because of its adjacency to the resonator at 5.55 GHz.

We performed a parametric study of the dependence of the charge-parity switching rate on the geometry to investigate the origin of the nonequilibrium quasiparticles in our devices. The qubit structure, typically a few hundred microns in size, can be a good antenna^19^, channelling stray photons at a few hundred gigahertz to the junction. The entire structure can be regarded as a pair of folded slots (Supplementary Material)^35^ (Fig. 3a, left) that support multiple resonant modes, determined primarily by the length of the long edges *L* of the metallic pads. For the fundamental mode, *L* = *λ*/2, *λ* being the effective mode wavelength obtainable from the real part of the input impedance *Z*_rad_ calculated via finite-element electromagnetic field simulations (Fig. 3b). For a capped qubit with the same geometry, the radiator mode frequency is slightly redshifted because of the additional capacitance between the metallic cap and the qubit. Moreover, because the cap behaves as a floating ground plane located in close proximity to the radiator, the induced currents also contribute to the radiated field; the virtual currents located on the other side of the cap are out of phase with respect to the currents on the qubit (Fig. 3a, right), therefore cancelling each other and leading to near-zero radiated power. This explains the much smaller radiation impedance when the same qubit is capped (Fig. 3b). The real part of the input impedance is directly related to the power transfer efficiency of the pair-breaking photons^19^:

2 $${e}_{{{{{{{{\rm{c}}}}}}}}}(\;f)=\frac{4{{{{{{{\rm{Re}}}}}}}}[{Z}_{{{{{{{{\rm{rad}}}}}}}}}]{{{{{{{\rm{Re}}}}}}}}[{Z}_{{{{{{{{\rm{J}}}}}}}}}]}{|{Z}_{{{{{{{{\rm{rad}}}}}}}}}+{Z}_{{{{{{{{\rm{J}}}}}}}}}{|}^{2}},$$

where *Z*_J_ is the junction impedance.

Figure 3c shows the parity switching rate Γ_*P*_ for qubits with different pad lengths, exhibiting a monotonic increase with the pad length *L* by two orders of magnitude for the uncapped qubits. With larger *L*, the frequency of the fundamental mode is reduced from 500 GHz (*L* = 80 μm) to 160 GHz (*L* = 300 μm), approaching twice the superconducting gap frequency f^*^ = 2Δ/*h*. Consequently, the power transfer efficiency at this critical frequency is enhanced. Note that for *L* = 240 μm, Γ_*P*_ ≈ 30s^−1^, in agreement with the switching rate measured for a device having similar *E*_J_/*E*_C_ ratio (20–30)^18^. Assuming the linear relation Γ_*P*_ = *γ* *e*_c_(f^*^), where *γ* is a constant indicating the conversion efficiency between the incoming photon flux (excluding the geometry-dependent factor *e*_c_) and the observed parity switching events, we find that *γ* = 3 × 10^5^s^−1^ gives the best agreement between the experiments and simulation predictions. This value of *γ* is used in all cases, since they share a nominally identical setup. For the capped qubits with a similar size, Γ_*P*_ is approximately an order of magnitude lower, consistent with the predictions.

The above result supports the hypothesis that pair-breaking photons absorbed by the antenna mode are responsible for the excessive quasiparticles in our devices; it also validates our method of protecting qubits from stray photons via capping, which is predicted to be effective across different regimes, from *E*_C_ > 1 GHz (charge qubits) to *E*_C_ < 0.3 GHz (transmon qubits). The parity switching time *T*_*P*_ for the low energy states of transmon qubits, while not detectable because of their insensitivity to charge, is estimated to be 10–100 ms (see Fig. 3c). Since *T*_*P*_ can set an upper limit on the qubit coherence times^36^, it is important to prevent nonequilibrium quasiparticles from compromising the qubit performance, especially with the state-of-the-art coherence time of transmon qubits approaching the millisecond mark^37,38^.

We also investigated the dependence of the charge-parity switching rate on the pad-to-ground distance *d* (the aperture size, see Fig. 1b). The experimental result again showed good agreement with predictions using the same method and parameters as before (Fig. 3d). Enlarging the gap between the pads and the ground plane increases the effective wavelength *λ* of the fundamental mode, giving rise to larger Γ_*P*_ values (Supplementary Material).

In addition to parity switching, we observed significantly improved offset charge stability. The charge offset for all six qubits on a single chip was monitored continuously and simultaneously via repeated spectral scans (Fig. 3e). The occurrence of discrete charge jumps (jump amplitude > 0.1*e*) was ~1–9 times over 40 h (Fig. 3f), corresponding to an offset charge jump rate of 0.007–0.07 mHz; the accumulated offset deviation during the long-term drift was within 1*e*, a significantly less volatile result than those previously observed (jump rate ~ 1.35 mHz)^4^. This difference can be explained by the reduced scattering cross section inside the substrate of our devices, which have smaller capacitor pads and a floating design (Supplementary Material). However, differences in the fabrication materials may be another important factor, as suggested recently in ref. 39, where an average jump rate of ~0.7 mHz was measured. Note that we do not observe simultaneous jumps between different qubits. Considering the relatively small footprint of our qubits and their separation (1.3 mm), this is consistent with the previous observation of charge jumps being correlated for qubits separated by up to 0.64 mm but not if separated by 3 mm^4^.

We also measured the coherence properties of the qubits. As shown in Fig. 3g, the energy relaxation rates Γ_1_ of the charge qubit (Q1) measured at different offset biases (different qubit frequencies) are relatively uniform, with slight variations between 10 μs and 25 μs, comparable to that of our regular transmon qubits fabricated using similar processes. The spin-echo pure-dephasing rates Γ_ϕ_ are in good agreement with a low-frequency, presumably 1/*f*-type, charge-noise model. The extracted noise spectral density is ~${(2.0\times 1{0}^{-3}e)}^{2}$ at 1 Hz, in line with charge noise observed in other experiments^40,41^.

### Effect of temperature on parity switching

**Fig. 4:**
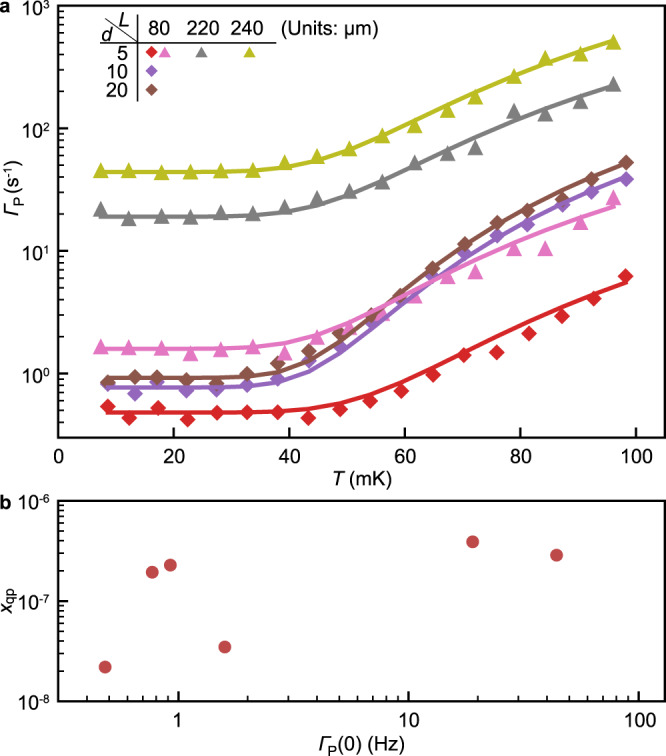
Effect of temperature on parity switching. **a** Parity switching rates measured at *n*_g_ = 0 as a function of the nominal mixing chamber temperature *T* for several uncapped qubits with different *L* and *d*, whose combinations are colour-coded. Symbols identify different samples. The solid lines are fit to Eq. (3). All curves share the same value for the superconducting gap in the pads (2Δ_0_/*h* = 87 GHz) and in the junction leads (2Δ/*h* ≃ 99 − 105 GHz (Supplementary Material)) on a given chip. The only remaining free parameter for a single qubit is the normalized quasiparticle density *x*_qp_. **b** Extracted quasiparticle density *x*_qp_ plotted versus the low-temperature parity switching rate Γ_*P*_(0).

Next, we investigated how temperature affects the parity switching rate. After intentionally heating the mixing chamber, we measured the temperature dependence of Γ_*P*_ at *n*_g_ = 0 for qubits with different *L* and *d* (Fig. 4a). At low temperatures, Γ_*P*_(*T*) did not display any clear temperature dependence and fluctuated around a qubit-specific average value Γ_*P*_(0) (up to these fluctuations, Γ_*P*_(0) agrees with the data in Fig. 3c and d); Γ_*P*_(*T*) then started to increase with the temperature near 40 − 60 mK.

There is an energy difference of ~30 μeV, equivalent to 350 mK, between the superconducting gaps in the pads (Δ_0_) and in the junction strips (Δ) because of the unequal aluminium film thicknesses (Fig. 1e). Therefore, at temperatures well below this value the pads act as quasiparticle traps^20^. As the temperature increases, the quasiparticles can be thermally excited from the pads to the strips and hence reach the junction, which could explain the increase in Γ_*P*_(*T*) with temperature starting near 50 mK.

To quantify the above consideration, we can relate the parity switching rate to the normalized density of the quasiparticles in the pads *x*_qp_ ≪ 1 (see Supplementary Note 8 (Supplementary Material) for details):

3 $${{{\Gamma }}}_{P}(T)={{{\Gamma }}}_{P}(0)+\frac{16{E}_{{{{{{{{\rm{J}}}}}}}}}}{{{\Delta }}}{c}_{0}^{2}\frac{{\epsilon }_{0}}{h}{e}^{\frac{-({{\Delta }}-{{{\Delta }}}_{0})}{{k}_{{{{{{{{\rm{B}}}}}}}}}T}}{x}_{{{{{{{{\rm{qp}}}}}}}}}\sqrt{\frac{{{{\Delta }}}_{0}}{2\pi {k}_{{{{{{{{\rm{B}}}}}}}}}T}}F\left(\frac{{\epsilon }_{0}}{2{k}_{{{{{{{{\rm{B}}}}}}}}}T},\frac{{k}_{{{{{{{{\rm{B}}}}}}}}}T}{2{{\Delta }}}\right),$$

where Γ_*P*_(0) accounts for all possible temperature-independent contributions, *ϵ*_0_ is the energy difference between $\left|{{{{{{{\rm{{g}}}}}}}^{E}}}\right\rangle$ and $\left|{{{{{{{\rm{{g}}}}}}}^{O}}}\right\rangle$, ${c}_{0}=|\left\langle {{{{{{{\rm{{g}}}}}}}^{E}}}\right|\cos (\hat{\frac{\phi }{2}})\left|{{{{{{{\rm{{g}}}}}}}^{O}}}\right\rangle|$ is the tunnelling matrix element between them, and the function $F(x,y)=\cosh (x)[{K}_{1}(x)-xy{K}_{0}(x)]$ (with *K*_*i*_ being the modified Bessel function of the second kind). For each qubit, *ϵ*_0_ and *c*_0_ can be evaluated using *E*_J_ and *E*_C_ values obtained from previous measurements. We assume the same Δ for all qubits on a given chip and simultaneously fit the data in Fig. 4a to Eq. (3). We obtain f^*^ = 2Δ/*h* between 99 GHz and 105 GHz (depending on the chip), which is consistent with a 30 nm aluminium thin film^32^. The quasiparticle density *x*_qp_ is the only adjustable parameter available to fit the temperature effect for a given qubit and Fig. 4b shows the extracted *x*_qp_ as function of Γ_*P*_(0). Qualitatively, the increase in density with the parity switching rate indicates that pair breaking at the junction (followed by diffusion to and trapping in the pads) is a significant source of quasiparticles, with the steady-state nonequilibrium density determined by the balance between generation and recombination. Therefore, although our measurements of Γ_*P*_ cannot directly distinguish between parity switching due to photon-assisted tunnelling or due to quasiparticles generated by other mechanisms, such as high-energy impacts of elevated temperature, the study of the temperature dependence enables us to conclude that these mechanisms, if present, are not the main sources of parity switching at low temperature (see Supplementary Note 8 (Supplementary Material) for more details).

## Discussion

Our characterization of several tens of superconducting qubits with extended parameter regimes indicates that pair breaking at the junction by stray photons of sufficient energy is the main mechanism responsible for parity switching as well as the generation of excess quasiparticles. This mechanism is local and affects each qubit independently. Because the parity switching rate Γ_*P*_ can be reduced by one to two orders of magnitude with simple engineering of the device geometry, e.g., using the capping technique and reducing the qubit footprint, novel miniaturized qubit designs^42–44^ may be advantageous in this regard. Meanwhile, further studies are required to evaluate the quasiparticle trapping effectiveness of the lower-gap capacitor pads and to optimize their design.

Finally, note that in our experiment we found no evidence of correlated increases in the parity switching rates of different qubits during simultaneous parity monitoring (similar to the simultaneous charge offset monitoring shown in Fig. 3e). This is in contrast to the correlated frequency shifts measured in resonators^3^ or the correlated increase of the relaxation errors in qubits^5^. This suggests that our floating design with a ground plane (similar to the phonon traps of ref. 13) and superconducting traps may also effectively suppress correlated errors, as suggested in ref. 45.

## Methods

### Device fabrication and measurement setup

The devices are made in a two-step process on c-plane sapphire wafers. In the first step, a layer of 100-nm-thick aluminium is deposited on the sapphire substrate at a growth rate of 1 nm/s with a base pressure of 10^−10^ Torr. The base metal is patterned with photolithography and subsequent dry etching using BCl_3_/Cl_2_. In the second step, the Josephson junctions are made in the Manhattan style using the double-angle evaporation to form the Al/AlO_x_/Al stack. The thickness of the first and second aluminium film is about 30 nm and 40 nm respectively. After ion milling, a final 200 nm-thick aluminium layer is deposited for making the bandage. For the flip-chip sample, the fabrication processes are identical. The two single-sided sapphire dies are bonded together using four rectangular spacers (2 mm × 2 mm × 10 μm) made of SU-8 photoresist at the corners.

The samples are mounted inside a BlueFors LD400 dilution refrigerator at a nominal base temperature <10 mK. In our standard setup, the sample is protected by a aluminium or copper holder box, a *μ*-metal shield, a few layers of copper and aluminium shields, and an outer *μ*-metal shield. Infra-red filters are used in all control and readout lines. These measures help block stray photons from reaching the sample via open space and cables. More details can be found in Supplementary Note 2.

### Charge-parity monitor

For qubits with small *E*_J_/*E*_C_ ratio, the difference between the g-e transition frequencies of even and odd parity at *n*_g_ = 0 is relatively large (typically a few GHz). This leads to very different resonator frequencies due to strong qubit-resonator coupling. Utilizing the difference in resonator response, we send a probe tone at the resonator frequency of certain parity to detect the parity. The measurement is done either with pulsed signals generated from an AWG and collected by a digitizer, or with continuous signals using a network analyzer. In the pulsed case, the probe pulse is typically 10 μs long and repeated every 0.3 ms. The single-shot result—99.14% fidelity for parity classification—is smoothed by taking a moving average to remove noise from thermal and measurement-induced excitation (see Supplementary Note 4 for more details).

For qubits with larger *E*_J_/*E*_C_ ratio (20 ~ 30), the frequency discrepancy between different parities becomes small (0.1 ~ 1 MHz) leading to unnoticeable difference in resonator frequency. Instead of direct dispersive readout, we use the Ramsey-type parity monitor as introduced in ref. 34 for parity detection. In the Ramsey experiment, we set the carrier frequency of the *π*/2-pulses at ${\omega }_{{{{{{{{\rm{drive}}}}}}}}}=({\omega }_{{{{{{{{\rm{ge}}}}}}}}}^{{{\rm{E}}}}+{\omega }_{{{{{{{{\rm{ge}}}}}}}}}^{{{{{{{{\rm{O}}}}}}}}})/2$ and the free-evolution time between the pulses to $\tau=\pi /2({\omega }_{{{{{{{{\rm{ge}}}}}}}}}^{{{\rm{E}}}}-{\omega }_{{{{{{{{\rm{ge}}}}}}}}}^{{{{{{{{\rm{O}}}}}}}}})$. Under such a pulse sequence, the qubit evolves to the excited (ground) state for even (odd) parity, allowing us to differentiate parity state. The sequence is typically repeated every 0.1 ms.

## Supplementary information

Supplementary Information

## Data Availability

All data obtained in the study are available from the corresponding author upon reasonable request.
