# Supplementary material for: Engineering superconducting qubits to reduce quasiparticles and charge noise
Source: Nat Commun. 2022 Nov 23;13:7196. doi: 10.1038/s41467-022-34727-2 (PMC9684549; doi:10.1038/s41467-022-34727-2)
Supplement: Supplementary file 1 — Supplementary Information [file 41467_2022_34727_MOESM1_ESM.pdf]

Supplementary information: Engineering superconducting qubits to reduce quasiparticles and charge noise

Xianchuang Pan,[1,2,3,*] Yuxuan Zhou,[1,2,3,4,*] Haolan Yuan,[1,2,3,4] Libo Zhang,[1,2,3] Jian Li,[1,2,3] Song Liu,[1,2,3] Zhi Hao Jiang,[5] Gianluigi Catelani,[6,7,†] Ling Hu,[1,2,3,‡] Fei Yan,[1,2,3,§] and Dapeng Yu[1,2,3,4]

[1] *Shenzhen Institute for Quantum Science and Engineering, Southern University of Science and Technology, Shenzhen, Guangdong, China*
[2] *International Quantum Academy, Shenzhen, Guangdong, China*
[3] *Guangdong Provincial Key Laboratory of Quantum Science and Engineering, Southern University of Science and Technology, Shenzhen, Guangdong, China*
[4] *Department of Physics, Southern University of Science and Technology, Shenzhen, Guangdong, China*
[5] *State Key Laboratory of Millimeter Waves, School of Information Science and Engineering, Southeast University, Nanjing, China*
[6] *JARA Institute for Quantum Information (PGI-11), Forschungszentrum Jülich, 52425 Jülich, Germany*
[7] *Quantum Research Centre, Technology Innovation Institute, Abu Dhabi, UAE*

SUPPLEMENTARY NOTE 1: DEVICE DESIGN AND FABRICATION

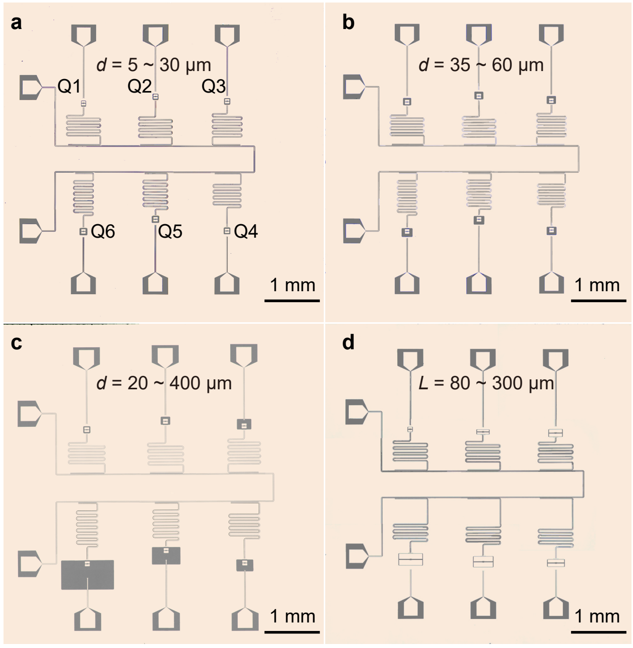

Supplementary Figure 1: Optical micrograph of planar samples (S1∼S4). Dark: exposed sapphire substrate; light: base aluminium layer. **a**, Sample S1, with variation of pad-to-ground distance d from 5 to 30 μm. **b**, Sample S2, with d from 35 to 60 μm. **c**, Sample S3, with d from 20 to 400 μm. **d**, Sample S4, with the pad length L from 80 to 300 μm.

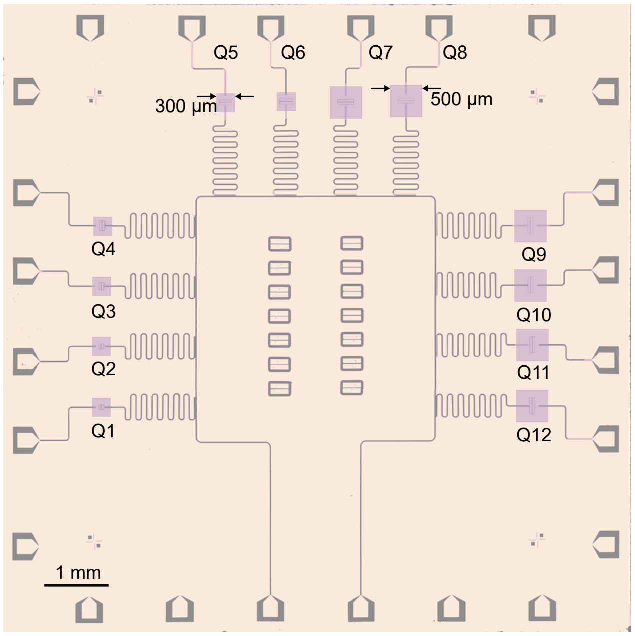

Supplementary Figure 2: Optical micrograph of the flip-chip sample S8 (bottom chip). The 12 qubits differ by the pad length L and E_J (Supplementary Table I). Purple squares indicate the metallic caps on the top chip covering each of the 12 qubits. The caps have two different size, 300 μm and 500 μm. All qubits share the same pad width $W = 35$ μm and pad-to-ground distance $d = 5$ μm.

Data presented in the main text are taken from five planar sample chips and one flip-chip sample. Each planar sample hosts 6 qubits with varying geometries as shown in Supplementary Fig. 1. The device parameters are listed in Supplementary Table I. Data used to investigate the dependence of the parity switching rate on pad-to-ground distance d (Fig. 3d in the main text) are from sample S1∼S3; data used to investigate the dependence on pad length L (Fig. 3c in the main text) are from sample S4∼S5 (same design). Data shown in Fig. 2 of the main text are from the qubit S1−Q1. Data shown in Fig. 4 of the main text are from sample S1, S4,

* These authors have contributed equally to this work.
† g.catelani@fz-juelich.de
‡ hul@sustech.edu.cn
§ yanf7@sustech.edu.cn

Device-Qubit	L (μm)	W (μm)	d (μm)	E_{J}/h (GHz)	E_{C}/h (GHz)	$\frac{E_{\mathrm{J}}}{E_{\mathrm{C}}}$	$\omega_{\mathrm{ge}}^{\max}/2\pi$ (GHz)	$\omega_{\mathrm{ge}}^{\min}/2\pi$ (GHz)	$g/2\pi$ (MHz)	$\omega_{\mathrm{r}}/2\pi$ (GHz)	T_1 (μs)	T_ϕ (μs)	T_P (s)	holder material	cap (μm)	CR110	Fig.
S1−Q1	80	35	5	4.67	1.40	3.34	6.833	4.473	24.3	5.556	24.4	22.8	1.918				2, 4
S1−Q2	80	35	10	4.27	1.48	2.89	6.954	4.135	20.4	5.607	29.4	13.4	1.235				3d∼g
S1−Q3	80	35	15	4.45	1.52	2.93	7.191	4.289	22.5	5.712	16.7	14.1	1.159	Al	no	yes	S5a
S1−Q4	80	35	20	4.63	1.53	3.03	7.268	4.469	23.3	5.753	25.8	20.9	0.987				S7∼9
S1−Q5	80	35	25	4.52	1.54	2.94	7.255	4.364	22.6	5.830	22.0	22.3	1.172				S18
S1−Q6	80	35	30	4.59	1.54	2.98	7.325	4.414	22.7	5.883	23.8	3.0	0.761				S21∼23
S2−Q1	80	35	35	–	–	–	–	–	–	5.552	–	–	0.691				
S2−Q2	80	35	40	–	–	–	–	–	–	5.610	–	–	0.531				
S2−Q3	80	35	45	–	–	–	–	–	–	5.713	–	–	0.541	Cu	no	yes	3d
S2−Q4	80	35	50	–	–	–	–	–	–	5.756	–	–	0.431				
S2−Q5	80	35	55	–	–	–	–	–	–	5.831	–	–	0.320				
S2−Q6	80	35	60	–	–	–	–	–	–	5.882	–	–	0.353				
S3−Q1	80	35	20	3.23	1.49	2.17	6.585	3.126	15.7	5.554	54.2	76.9	1.123				
S3−Q2	80	35	40	3.36	1.51	2.23	6.729	3.279	15.5	5.609	42	400.0	0.673				
S3−Q3	80	35	80	3.24	1.52	2.13	6.728	3.130	13.2	5.712	–	–	0.175	Al	no	yes	3d
S3−Q4	80	35	100	3.47	1.51	2.30	6.806	3.386	14.3	5.754	–	–	0.244				S4b
S3−Q5	80	35	200	–	–	–	–	–	–	5.833	–	–	0.164				
S3−Q6	80	35	400	–	–	–	–	–	–	5.885	–	–	0.251				
S4−Q1	80	35	5	7.61	1.30	5.87	7.845	6.754	46.5	5.521	–	–	2.268				
S4−Q2	220	35	5	6.92	0.78	8.89	5.774	5.505	4.7	5.581	–	–	0.074				
S4−Q3	240	60	5	12.25	0.44	28.09	6.068	6.067	–	5.634	4.4	7.2	0.030	Al	no	yes	3c
S4−Q4	300	60	5	15.31	0.40	38.66	6.5402	6.5401	–	5.700	12.3	14.0	0.016				S5b,d
S4−Q5	350	90	5	–	–	–	–	–	–	5.756	–	–	–				S23
S4−Q6	420	100	5	–	–	–	6.2184	6.2184	–	5.800	–	–	–				
S5−Q1	80	35	5	6.25	1.31	4.77	7.246	5.740	50.0	5.515	–	–	0.610				
S5−Q2	220	35	5	5.12	0.76	6.73	4.907	4.400	119.1	5.596	–	–	0.046				3c
S5−Q3	240	60	5	14.66	0.37	39.62	6.2013	6.2012	–	5.628	–	–	0.022	Al	no	yes	4
S5−Q4	300	60	5	11.99	0.43	27.88	5.9853	5.984	–	5.695	8.6	45.7	0.011				S5d
S5−Q5	350	90	5	–	–	–	6.061	6.061	–	5.752	13.1	28.3	–				S23
S5−Q6	420	100	5	–	–	–	5.8345	5.8345	–	5.799	16.1	22.6	–				
S6−Q1∼Q6	80	35	20	3.50	1.40	2.50	6.558	3.257	16.1	5.703	5	9	0.374	Al	no	no	S4a S20
S7−Q1	80	35	20	2.79	1.43	1.95	6.214	2.774	11.25	5.556	–	–	0.940				
S7−Q2	80	35	40	–	–	–	–	–	–	–	–	–	0.610				
S7−Q3	80	35	80	–	–	–	–	–	–	–	–	–	0.072	Al	no	no	S4b
S7−Q4	80	35	100	–	–	–	–	–	–	–	–	–	0.074				
S7−Q5	80	35	200	–	–	–	–	–	–	–	–	–	0.089				
S7−Q6	80	35	400	–	–	–	–	–	–	–	–	–	0.140				
S8−Q2	80	35	5	2.35	1.29	1.82	5.566	2.281	16.9	4.611	55.3	3.1	2.006		300		
S8−Q3	120	35	5	2.23	0.95	2.35	4.260	2.230	12.9	4.673	–	–	1.600		300		
S8−Q4	160	35	5	2.43	0.75	3.20	3.650	2.320	5.2	4.702	–	–	1.006		300		3c
S8−Q5	180	35	5	4.45	0.66	6.74	4.254	3.858	19.2	4.733	39.2	–	0.904	Al	300	yes	S19
S8−Q6	210	35	5	4.71	0.59	7.97	4.104	3.899	18.6	4.770	–	–	0.615		300		S23
S8−Q7	260	35	5	4.84	0.49	9.92	3.832	3.712	18.0	4.805	13.8	25.1	0.603		500		
S8−Q8	260	35	5	5.79	0.46	12.58	4.116	4.059	23.0	4.835	35.3	19.3	0.655		500		
S9−Q1	80	35	20	–	–	–	–	–	–	5.850	–	–	0.511				
S9−Q2	80	35	50	–	–	–	–	–	–	5.800	–	–	0.249				
S9−Q3	80	35	80	–	–	–	–	–	–	5.751	–	–	0.146	Al	no	no	S5c
S9−Q4	80	35	110	–	–	–	–	–	–	5.750	–	–	0.196				
S9−Q5	80	35	140	–	–	–	–	–	–	5.700	–	–	0.157				
S9−Q6	80	35	170	–	–	–	–	–	–	5.650	–	–	0.152				
S10−Q1	80	35	20	–	–	–	–	–	–	5.850	–	–	0.652				
S10−Q2	80	35	50	–	–	–	–	–	–	5.800	–	–	0.292				
S10−Q3	80	35	80	–	–	–	–	–	–	5.753	–	–	0.158	Al	no	no	S5c
S10−Q4	80	35	110	–	–	–	–	–	–	5.750	–	–	0.181				
S10−Q6	80	35	170	–	–	–	–	–	–	5.650	–	–	0.149				

Supplementary Table 1: Device parameters and setup. L, W, d are the pad length, pad width, and pad-to-ground distances. The listed T_1, T_ϕ and T_P are measured at the minimum qubit frequency at $n_{\mathrm{g}} = 0$. We also list the information about the sample holder material, the metallic cap size, the use of CR110 filter after the bias tee, and the figures the device is related to. Note that the six qubits in S6 have all the same design; the typical data listed here comes from S6-Q1. Sample S7 has the same design as S3. Sample S8 is the flip-chip device. The wiring or shielding setup of sample S6, S7, S9, S10 is different from the standard (optimal) one shown in Supplementary Fig. 3, so we did not include related data in the main text figure. For samples S2, S7, S9, S10, we only measured T_{P} data. The S10-Q5 qubit is broken. All the data in Figs. S7∼S9, S21∼S22 are from S1-Q1. Unmeasured parameters are left blank. Here, Fig. S# means Supplementary Fig. #.

and S5. There are two more planar samples (S6 and S7) considered in this supplement, see Supplementary Table I and Supplementary Note 2. The chips are either packaged in an aluminium or copper sample holder for testing. We do not observe appreciable differences in qubit performance between different holder materials and between different cooldowns with the same setup and wiring.

The devices are made in a two-step process on c-plane sapphire wafers. The first step is to pattern the base circuit. We deposited 100-nm-thick aluminum on c-plane sapphire substrate in a PLASSYS system at a growth rate of 1 nm/s with a base pressure of 10^{-10} Torr. After photolithography, we use BCl_3/Cl_2 etch in an inductively coupled plasma (ICP) dry etcher. In the second step, the junctions are fabricated in the bridge-free Manhattan style. We use double-angle evaporation to make the $Al/AlO_x/Al$ stack. The first aluminium film is about 30 nm thick and the second one is about 40 nm. Then the excess aluminium is lifted off with a stripper. An ion-milling step is performed before we add a final layer of aluminium film (200 nm thick), connecting the junction leads and capacitor pads in order to make direct galvanic contact.

For the flip-chip sample, the bottom chip, which host 12 qubits with varying geometries as shown in Supplementary Fig. 2, shares similar design and fabrication processes as the planar samples. Each qubit is covered by a metallic cap, a square-shaped aluminium pad, on the opposing die. The two single-sided sapphire dies are bonded together using four spacers (2 mm×2 mm) at the corners. The spacers made of SU-8 photoresist extend a vertical spacing of about 10 μm.

SUPPLEMENTARY NOTE 2: EXPERIMENTAL SETUP AND WIRING

The samples are mounted inside a BlueFors LD400 dilution refrigerator at a nominal base temperature of less than 10 mK; however, the excited-state population measured with regular transmon qubits [1] in the same setup gives a typical device or electronic temperature of 50-60 mK. The experimental setup is depicted in Supplementary Fig. 3. As shown in Supplementary Fig. 3, from the inside out, the device is protected by a aluminum or copper holder box, a μ-metal shield, a few layers of copper and aluminum shields, and an outer μ-metal shield. Control and readout signals are properly attenuated and filtered at multiple stages. At room temperature, we generally use a network analyzer for fast and cleaner data taking in the case of small parity switching rate $\Gamma_P < 100$ Hz. However, for large Γ_P, one has to use pulsed measurement based on a Ramsey-like sequence (see Supplementary Note 6) using an AWG-digitizer-based setup.

Empirically, we find two aspects in the setup that make a difference in the measured parity switching time. First, using a cryogenic μ-mental shield can help suppressing Γ_P by almost an order of magnitude, which suggests its effectiveness in radiation shielding (Supplementary Fig. 4, left); we also find it important to add an infrared (IR) filter to the common port of a bias tee (Supplementary Fig. 4, right), which is consistent with observations reported by other groups [2, 3]. Based on the above findings, we believe that the shield and IR filter are effective in blocking stray photons of about 100 GHz (or higher) from reaching the sample via open space and cables, respectively.

The photon flux seen by the device may vary depending

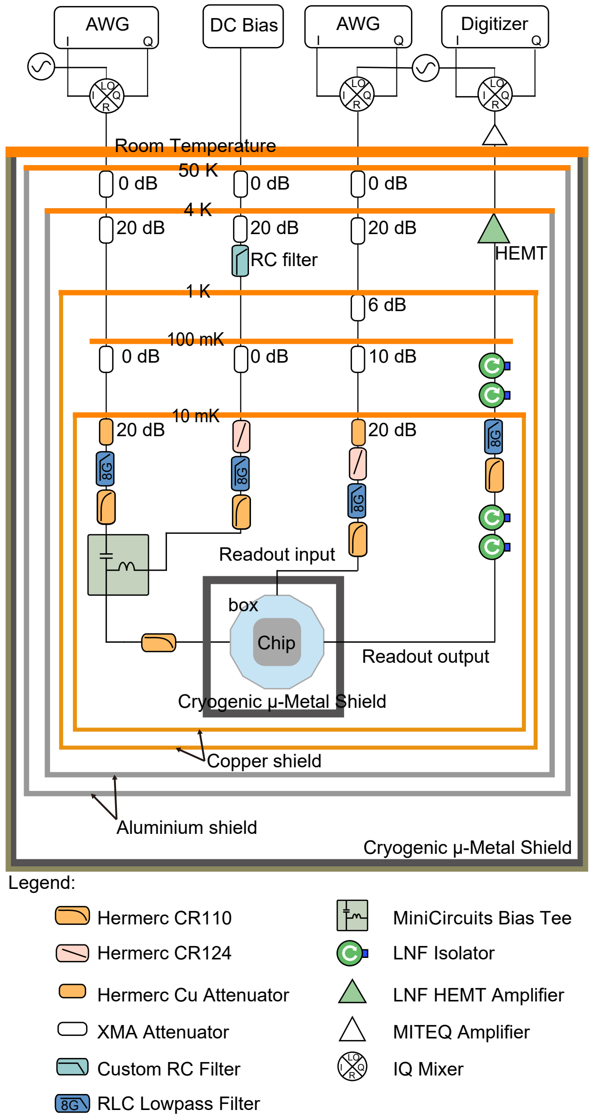

Supplementary Figure 3: Cryogenic wiring and signal synthesis at room temperature. We combine DC bias and RF signals at base temperature using a bias tee before sending them to the chip. All data shown in the main text is taken using this standard setup. A cryogenic μ-metal shield is used for shielding.

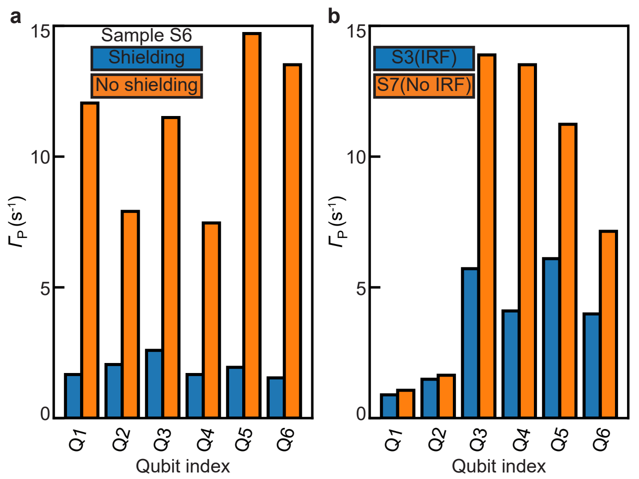

Supplementary Figure 4: Shielding and filtering. **a**, Comparison of Γ_P with or without the shield. The data is taken in different cooldowns using sample S6 with six identical qubits. There are no CR110 filters after the bias tee in both cases. **b**, Comparison of Γ_P with or without CR110 IR filter after the bias tee. The data is taken in the same cooldown from sample S3 (blue) and S7 (orange); the samples share the same design and, except for the IR filter, are measured with identical setups. There are μ-metal shields in both cases in **b**.

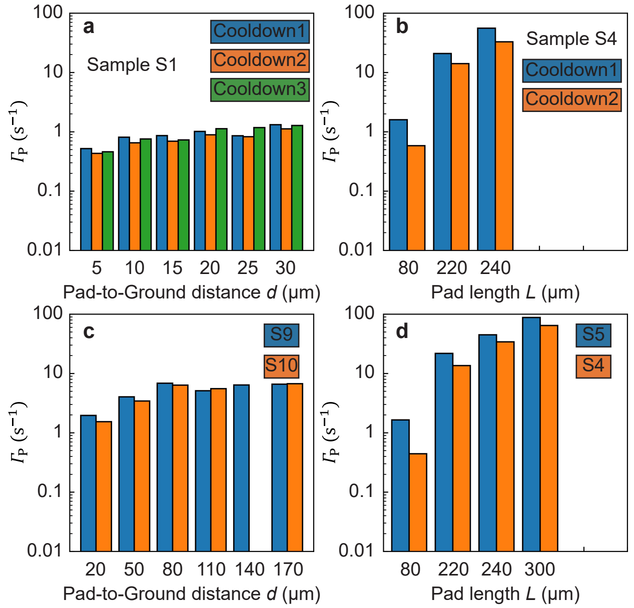

Supplementary Figure 5: Γ_P stability. a-b, Comparison of measured parity switching rates between different cooldowns for a given sample. **c-d**, Comparison of measured parity switching rates between samples of identical design during a given cooldown. The measurement setup is the same. Missing data is due to broken qubit or undetectable with our method (too small a dispersive shift).

on the specific filtering and shielding setup. For a nominally identical setup, We assume that the photon flux stays constant between different cooldowns. In Supplementary Fig. 5a∼b, we compare the parity switching rates measured in different cooldowns for two devices, which show good agreement to our assumption. The measured switching rates for a given device have relatively small change between different cooldowns (about a factor of 2 in the worst case). In addition, it can be seen that, over different cooldowns, the switching rates have consistent geometric dependence which can vary by one to two orders of manginitude, much greater than the random fluctuations between cooldowns. We also compare the parity switching rates of samples with identical design, meausured with an identical setup during a same cooldown as shown in Supplementary Fig. 5c∼d. The data also show consistent behavior with only small fluctuations. Therefore, we can safely assume that the qubits see a same amount of photon flux for a given shielding and filtering setup.

SUPPLEMENTARY NOTE 3: QUBIT SPECTROSCOPY

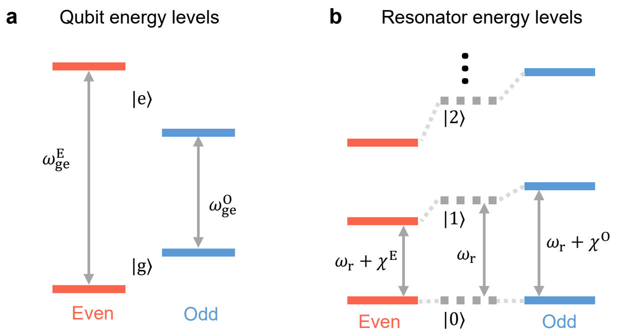

Supplementary Figure 6: Energy level diagram. **a**, Ground and excited states of a low-E_J/E_C qubit for even and odd parities. Parity switching events, associated with quasiparticle tunnelling or pair-breaking at the junction, exchange the two parities. In the shown example, the transition frequency between ground and excited states is higher (lower) in the even (odd) parity. **b**, Resonator levels in even and odd parity compared to the bare case (dashed grey). In the shown case, the resonator frequency is between the even-parity and odd-parity qubit frequency. The dressed resonator frequency in the even(odd) parity is red(blue)-shifted from its bare frequency (dashed).

For a typical qubit in the charge regime among our devices, Josephson and charging energies are in the range $E_J/h = 3.3$–4.6 GHz and $E_C/h = 1.4$–1.6 GHz ($E_J/E_C \sim 3$). As illustrated in Supplementary Fig. 6a, in such a case, energy levels are considerably different between different parities, leading to strong discrepancy in qubit transition frequencies, ω_{ge}^{E} and ω_{ge}^{O}, up to a few

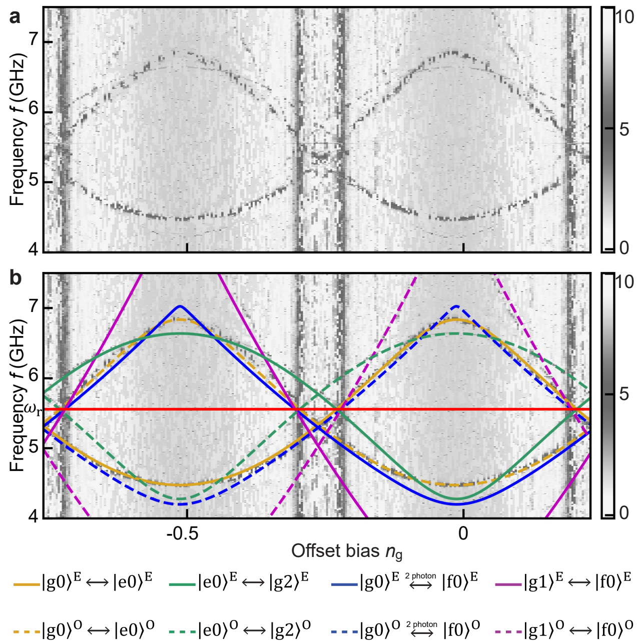

Supplementary Figure 7: Qubit spectroscopy with identified transitions. a, Qubit spectroscopy reproduced from Fig. 2b. **b**, Same as **a** with added coloured lines denoting various transitions. Solid (dashed) lines denote transitions in the even (odd) parity. The spectroscopy pulse is about 9 μs long. The sequence is repeated every 100 μs. Each datapoint presented is the average of 1000 repetitions.

GHz depending on the offset bias. In a circuit-QED architecture where the qubit is coupled to a resonator [4], such large discrepancy can result in appreciable difference in the dressed resonator frequency because of level repulsion. For example, Supplementary Fig. 6b shows the level diagram when the resonator frequency ω_{r} is between $\omega_{\mathrm{ge}}^{\mathrm{E}}$ and $\omega_{\mathrm{ge}}^{\mathrm{O}}$. The resonator frequency is pushed down (up) by $\chi^{\mathrm{E(O)}}$ ($\sim$1 MHz) when the qubit is in even (odd) parity. Similar to the dispersive measurement of the qubit state, sending a probe tone near the resonator frequency allows us to distinguish between different qubit parities, provided that the acquisition time is short compared to the average latching time between parity switches.

In Supplementary Fig. 7, we reproduce the qubit spectroscopy shown in Fig. 2 of the main text and identify all the visible transitions which include, for both parities, the g-e transition of the qubit ($|\mathrm{g0}\rangle - |\mathrm{e0}\rangle$), the two-photon g-f transition ($|\mathrm{g0}\rangle - |\mathrm{f0}\rangle$), the qubit-resonator sideband transitions ($|\mathrm{g1}\rangle - |\mathrm{f0}\rangle$ and $|\mathrm{e0}\rangle - |\mathrm{g2}\rangle$), and the resonator mode at ω_{r}.

SUPPLEMENTARY NOTE 4: CHARGE PARITY MONITOR

A. Parity detection with direct dispersive readout

To monitor charge parity evolution, we implement two different methods, one based on direct dispersive readout with the qubit in the ground state and one based on a conditional bit flip. For qubits with small $E_{\mathrm{J}}/E_{\mathrm{C}}$ ratio, the g-e transition frequencies at $n_{\mathrm{g}} = 0$ for even and odd parity are drastically different, leading to dissimilar resonator frequency. We utilize such parity-dependent resonator response and use direct dispersive readout for distinguishing parity. The measurement can be done either with pulsed probe signals generated with an AWG and collected by a digitizer, or with a network analyzer which probes continuously. In the pulsed case, the probe pulse is typically 10 μs long and repeated every 0.3 ms. The single-shot result – 99.14% fidelity for parity classification – is relatively noisy, but it can be smoothed by taking a moving average, see Supplementary Fig. 8a. The noise has two parts: small sampling noise scattered around one of the telegraph state and less frequent strong jumps to the excited state about which we shall discuss later.

In our data processing, we rotate the raw demodulated data in the complex plane and use the real part for subsequent analysis, Supplementary Fig. 8b. In the continuous case, since the probe pulse is effectively always-on, the sampling noise becomes much reduced and a clean telegraph signal can be obtained, Supplementary Fig. 8c. The power spectral density (Supplementary Fig. 8d) and the autocorrelation function (Supplementary Fig. 8e) – Fourier transform of power spectral density – measured with the two setups show good agreement, validating our measurement and analysis protocols.

Now we discuss about the origin of the random jump events shown in Supplementary Fig. 8a. In Supplementary Fig. 9a we plot the resonator response for qubit in four different states, $|\mathrm{g}\rangle^{\mathrm{O}}$, $|\mathrm{e}\rangle^{\mathrm{O}}$, $|\mathrm{g}\rangle^{\mathrm{E}}$, $|\mathrm{e}\rangle^{\mathrm{E}}$. It can be seen that $f_{\mathrm{r}}(|\mathrm{g}\rangle^{\mathrm{O}})$ is close to $f_{\mathrm{r}}(|\mathrm{e}\rangle^{\mathrm{E}})$, and $f_{\mathrm{r}}(|\mathrm{g}\rangle^{\mathrm{E}})$ is close to $f_{\mathrm{r}}(|\mathrm{e}\rangle^{\mathrm{O}})$. We can anticipate ambiguity between these pairs of states. We use a stronger probing amplitude for better pointer state separation in measurement associated with Supplementary Fig. 9b-e. Supplementary Fig. 9b shows an example time trace of repeated single-shot measurement where a parity switch is observed. From data collected from different sections of the trace, we can identify the corresponding state in the complex plane of the readout signal. First of all, $|\mathrm{g}\rangle^{\mathrm{O}}$ and $|\mathrm{g}\rangle^{\mathrm{E}}$ can be distinguished from each other by comparing Supplementary Fig. 9c and Supplementary Fig. 9d. Note that there is another small cluster adjacent to $|\mathrm{g}\rangle^{\mathrm{E}}$ which corresponds to $|\mathrm{e}\rangle^{\mathrm{O}}$, as can be confirmed by adding a π pulse at the odd-parity qubit frequency, which selectively prepares the $|\mathrm{e}\rangle^{\mathrm{O}}$ state. The result is shown in Supplementary Fig. 9e, where significantly more

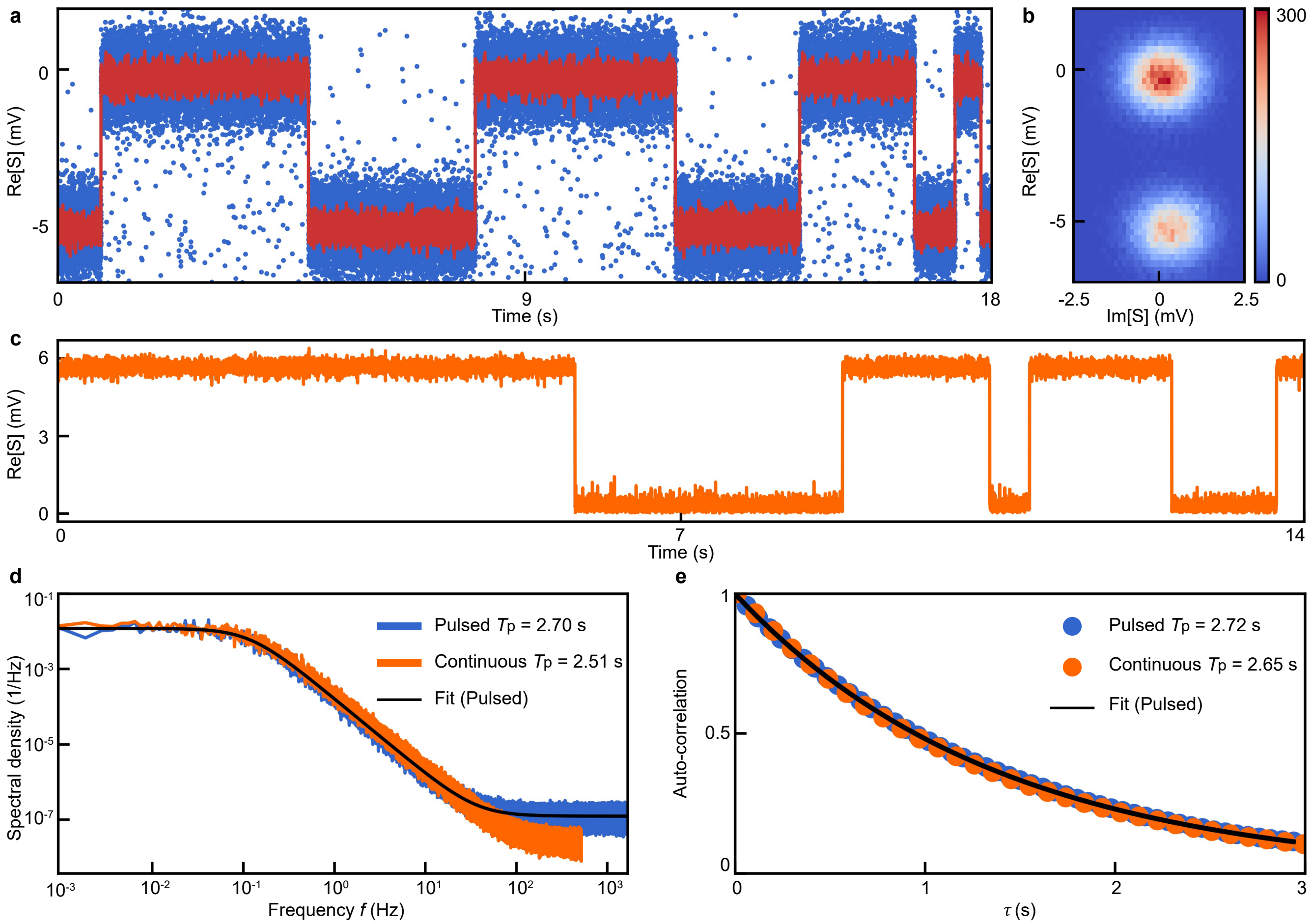

Supplementary Figure 8: Parity monitor with direct dispersive readout. **a**, Example of raw demodulated signal (blue dots) acquired in a pulsed measurement and its moving average (red line), calculated by taking the median of 10 consecutive points. A typical trace taken with the pulsed method is 18 s long with a time interval of 0.3 ms between points. **b**, Histogram of the raw complex data shown in **a**. **c**, Example of raw data acquired in a continuous measurement with a network analyzer. A typical trace is 14 s long with the time interval of 1 ms between points. **d**, Power spectrum of charge-parity fluctuations measured in pulsed (1200 repetitions) and continuous (1500 repetitions) measurement. We concatenate the repeated traces in (a,c) to extend the lower limit of the spectrum to 10^{-3} Hz. The background white noise is due to sampling noise which is stronger for the pulsed method, but does not influence the extracted T_P times. The black line is a Lorentzian plus white noise fit to the pulsed data. **e**, Normalized autocorrelation function $\langle P(0)P(\tau)\rangle$ of charge-parity fluctuations directly computed from the time-domain traces. The black line is an exponential fit to the pulsed data.

datapoints are present in the $|e\rangle^{O}$ cluster marked by the orange circle. Therefore, those orange points (identified from being inside the orange circle) in Supplementary Fig. 9b correspond to the case of the qubit being in the excited state and in the odd parity. Since $f_r(|g\rangle^{O})$ and $f_r(|e\rangle^{E})$ are even closer, the two clusters almost overlap with each other. We did not try to separate these two clusters. In a pulsed measurement setup, the data is taken with a digitizer; the acquisition time is usually a few microseconds (here, $3\,\mu$s) which is shorter than the typical T_1 time. Since we repeat every $100\,\mu$s, any residual excitation is most likely to be observed as a single jump event in the repeated time trace. On the other hand, in the continuous measurement done with network analyzer, the effective acquisition time (bandwidth ∼10 kHz) is usually much longer than T_1 so that the analyzer signal – being the averaged result – cannot resolve those excitation events.

There are two major reasons for the spurious excitation. First, there is thermal excitation due to finite device temperature. With low enough readout power, we typically observe an excitation level of 0.5% – 1%, equivalent to 50-60 mK temperature, consistent with other regular transmon qubits measured in the same setup. Then, there is also measurement-induced excitation. When we increase the amplitude of the measurement pulse as in Supplementary Fig. 9, we tend to see increased contrast between the two parities, as well

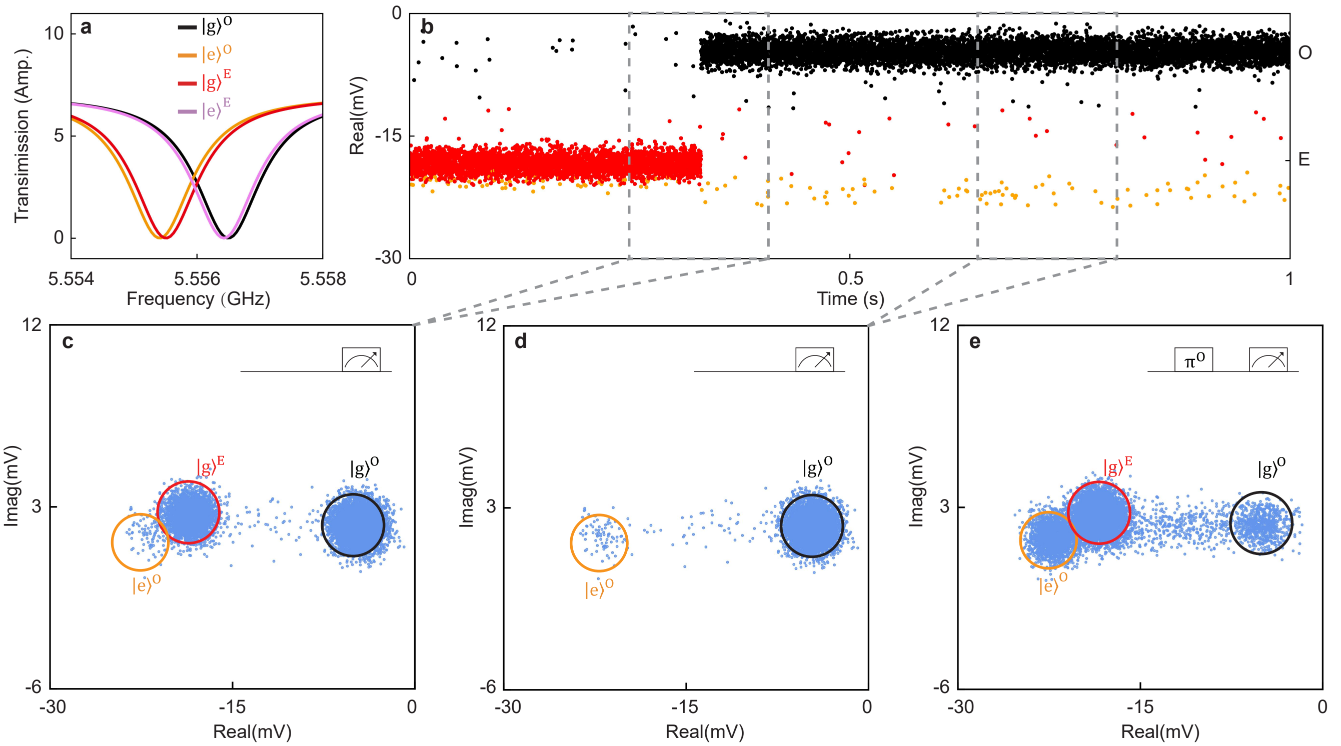

Supplementary Figure 9: Noise due to spurious qubit excitation induced by the probe signal. a, Readout resonator response for the four qubit states (ground/excited and even/odd) computed for sample S1-Q1. The resonant frequencies are $f_{\mathrm{r}}(|\mathrm{g}\rangle^{\mathrm{O}}) = 5.55650$ GHz, $f_{\mathrm{r}}(|\mathrm{g}\rangle^{\mathrm{E}}) = 5.55551$ GHz, $f_{\mathrm{r}}(|\mathrm{e}\rangle^{\mathrm{O}}) = 5.55541$ GHz, $f_{\mathrm{r}}(|\mathrm{e}\rangle^{\mathrm{E}}) = 5.55643$ GHz. **b**, An example of repeatedly measured single-shot time trace when the qubit is nominally prepared in the ground state. **c**, Demodulated signal amplitude plotted in the complex plane. Datapoints are taken from a section of time trace that samples both parities. **d**, Same as **c** but taken from a section that samples the odd parity only. **e**, Same as **c** but with a selective π pulse for the odd parity. Colored circles indicate the distribution for corresponding states. Data in this figure is taken with a stronger probe signal than in Supplementary Fig. 8.

as more frequent jumps to the other state.

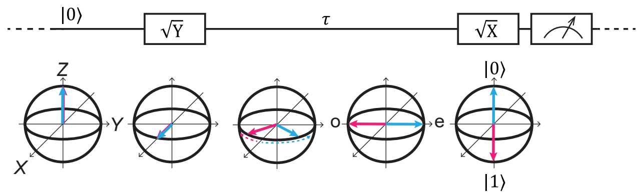

Supplementary Figure 10: Ramsey-based parity monitor. The sequence consists of two $\pi/2$ pulses separated by a waiting time τ, which is chosen as $\tau = \pi/2(\omega_{\mathrm{ge}}^{E} - \omega_{\mathrm{ge}}^{\mathrm{O}})$, such that the odd and even states are mapped to ground and excited qubit states, respectively, at the end of the sequence. The time τ is pre-calibrated before repeated measurements.

B. Parity detection with Ramsey sequence

For qubits with larger $E_{\mathrm{J}}/E_{\mathrm{C}}$ ratio (20∼30), the frequency discrepancy between different parities (0.1∼1 MHz) is exponentially suppressed in this ratio, and we can no longer use direct dispersive readout to distinguish between the parities. Instead, we use the Ramsey-type parity monitor as introduced in Ref. [5] and depicted in Supplementary Fig. 10. In the Ramsey experiment, we set the microwave drive frequency at $\omega_{\mathrm{drive}} = (\omega_{\mathrm{ge}}^{E} + \omega_{\mathrm{ge}}^{\mathrm{O}})/2$ and the free-evolution time $\tau = \pi/2(\omega_{\mathrm{ge}}^{E} - \omega_{\mathrm{ge}}^{\mathrm{O}})$. Such a detuning setting transforms the qubit to the excited (ground) state for even (odd) parity, which is a conditional bit flip and enables us differentiate parity state from qubit state measurements. The sequence is typically repeated every 0.1 ms.

SUPPLEMENTARY NOTE 5: ANTENNA MODE OF THE TRANSMON QUBIT

The transmon qubit structure, which has a typical size of a few hundred microns, can be an efficient receiving antenna, transferring photons of a few hundred GHz to the junction [6]. The absorbed photons at the junction can break Cooper pairs that tunnel through the barrier, giving rise to quasiparticle poisoning which

is detectable as parity switching. Aided by finite-element electromagnetic simulations, we validate the photon absorption model and provide a semi-quantitative explanation for the experimental observations.

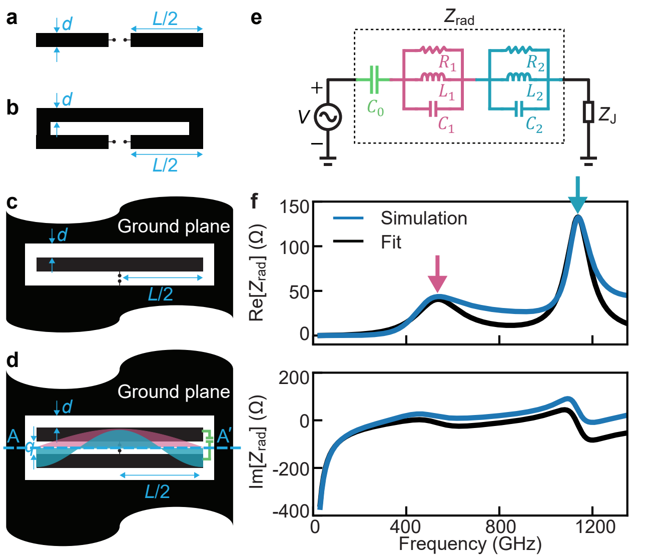

Supplementary Figure 11: Antenna mode of the qubit and equivalent circuit model. **a**, A simple half-wave dipole radiator. **b**, A folded dipole radiator obtained by folding and connecting the two dipole ends in a. **c**, A folded slot, which is the dual structure of b. **d**, A paired folded slot radiator, obtained by mirroring c about the line AA', which shares a same structure as a symmetric floating transmon qubit. **e**, Equivalent circuit of the qubit structure. Z_{rad} is the radiation impedance and Z_{J} is the impedance of Josephson junction. **f**, Real and imaginary part of Z_{rad} from finite-element simulation and from calculating the equivalent circuit in **e**. Arrows indicate the corresponding resonance modes. In this example, the qubit parameters are $L = 80\,\mu$m, $W = 35\,\mu$m and $d = 5\,\mu$m. In the equivalent circuit, $C_0 = 15$ fF, $R_1 = 40\ \Omega$, $L_1 = 5.3$ pH, $C_1 = 17$ fF, $R_2 = 130\ \Omega$, $L_2 = 2$ pH, $C_2 = 10$ fF.

A. Antenna mode of the qubit and equivalent circuit

From the perspective of antenna theory, the floating qubit structure used in our work can be thought of as evolved from a simple dipole radiator. As shown in Supplementary Fig. 11a, we start with a conventional half-wave dipole containing two metallic arms, each of length $L/2$, and a feed at the center. The fundamental mode frequency is found equating L to a half wavelength, while the higher-order resonances occur at frequencies such that L corresponds to an integer multiple of a half-wavelength. By folding the two ends of the dipole back around and electrically connecting them together to form a loop, a folded dipole radiator can be obtained (Supplementary Fig. 11b), where the transverse length L is the same. The input impedance of the folded dipole can be expressed as

$$Z_{\mathrm{fd}} = 4Z_{\mathrm{t}}Z_{\mathrm{d}}/(Z_{\mathrm{t}} + 2Z_{\mathrm{d}})\,, \tag{1}$$

where Z_{d} is the input impedance of a conventional dipole and Z_{t} is the input impedance of the transmission line formed by each folded arm with a short circuit loading [7]. At the fundamental mode resonant frequency, $Z_{\mathrm{fd}} = 4Z_{\mathrm{d}}$ since Z_{t} approaches infinity. In order to adapt such radiating structures to superconducting qubits based on coplanar waveguide circuits, the Babinet's principle is applied to the folded dipole to obtain its dual structure, i.e. a folded slot (Supplementary Fig. 11c). The input impedance of this dual radiating structure can be related to that of the folded dipole radiator as $Z_{\mathrm{fs}} = Z_0^2/Z_{\mathrm{fd}}$, where Z_0 is the impedance of free space. Since the electric field within the folded slot is primarily perpendicular to the long edges of the slot, by utilizing image theory, another folded slot can be created by mirror reflection of the original one about the line AA', resulting in the paired folded slot radiator, which is structurally equivalent to a symmetric floating transmon (Supplementary Fig. 11d). It should be noted that the line AA' can be regarded as an electrical wall, such that the introduction of the additional folded slot below the line AA' will not affect the field distribution but increase the input impedance by a factor of two, i.e. $Z_{\mathrm{rad}} = 2Z_{\mathrm{fs}}$.

The frequency-dependent response of the radiation impedance (Z_{rad}) over an ultra-wide frequency range, e.g. from ∼DC (10 GHz) to 1.2 THz, of the paired folded slot radiator can be modeled by an equivalent circuit. In order to ensure the passivity and causality of impedance response of the radiator, the equivalent circuit is made of capacitors, inductors, and resistors connected in series and/or parallel [8]. Based on the structural characteristics of the pair folded slot radiator, it can be deduced that the fundamental resonance (mode 1) occurs at frequencies such that L equals to about a half wavelength and the first higher-order resonant mode (mode 2) is found at frequencies such that L is around a full wavelength. For slot radiators with the electric field polarized perpendicular to the long edges, the gap provides the capacitance while the metallic ground offers the inductance. Thus, both of the two slot modes can be modeled as a parallel RLC circuit with different circuit element values of C_i, L_i, and R_i, where $i = 1, 2$. In addition, there exists a coplanar capacitance, denoted as C_0, between the edges of the two metallic pads in the top and bottom folded slots. At frequencies away from the resonant modes, the two parallel RLC circuits possess a low impedance, behaving like a short circuit, indicating that these two RLC resonant circuits and the inter-pad capacitor should be connected in series. Hence, the equivalent circuit can be modeled as shown in Supplementary Fig. 11e and the corresponding Z_{rad} can

be experessed as

$$Z_{\rm rad} = \frac{1}{j\omega C_0} + \frac{1}{1/R_1 + j\omega C_1 + 1/j\omega L_1} + \frac{1}{1/R_2 + j\omega C_2 + 1/j\omega L_2}, \quad (2)$$

In order to validate this circuit model, we simulate the entire qubit structure using a finite-element electromagnetic field solver [9] to obtain $Z_{\rm rad}$. We use a similar method as presented in Ref. [6], which embeds the qubit layer in an uniform dielectric medium with effective permittivity $\epsilon_{\rm eff} = (1 + \epsilon_{\rm r})/2$ ($\epsilon_{\rm r} = 11$ for sapphire). The real and imaginary part of the obtained frequency responses of $Z_{\rm rad}$ are shown in Supplementary Fig. 11f for the example of a qubit with $L = 80\,\mu$m, $W = 35\,\mu$m, and $d = 5\,\mu$m, and are fitted using the equivalent circuit model from Eq. (2); good agreement can be observed over a frequency range from near DC (10 GHz) to over 1.35 THz, thus validating the proposed circuit model for this physical transmon structure. At low frequencies well below any resonance of the radiator, $Z_{\rm rad}$ should exhibit a purely capacitive response, as shown in the imaginary part of $Z_{\rm rad}$. The value of C_0 extracted from the simulation corresponds to a charging energy $E_{\rm C}/h \simeq 1.36\,$GHz, comparable to that estimated from qubit spectroscopy, see Supplementary Table I. As frequency increases to the fundamental resonance, because the input impedance of the transmission line $Z_{\rm t} \to \infty$ and the dipole impedance $Z_{\rm d}$ has a large value right at resonance frequency, the folded dipole, $Z_{\rm fd} \approx 4Z_{\rm d}$ according to Eq. (1), has a large impedance. Therefore, the corresponding folded slot, $Z_{\rm fs} = 2Z_0^2/Z_{\rm fd}$, has a low impedance, as can be observed from the lower peak of the fundamental mode in the real part of $Z_{\rm rad}$ in Supplementary Fig. 11f. At the frequency of the first higher-order resonant mode, a large $Z_{\rm rad}$ can be found since $Z_{\rm t}$ approaches zero when the value of L is about a full wavelength.

Next, we use the radiation impedance to estimate how efficiently pair-breaking photons are absorbed at the junction through the qubit antenna. In the equivalent circuit, we model the external radiation as a Thévenin-equivalent generator with a voltage V [10]. The qubit structure apart from the junction can be seen as the internal impedance of the generator, which is $Z_{\rm rad} = R_{\rm rad} + jX_{\rm rad}$. The junction is the load with an impedance $Z_{\rm J} = R_{\rm J} + jX_{\rm J}$. The current is then $I = V/(Z_{\rm rad} + Z_{\rm J})$, and the power delivered to the antenna terminals $P_{\rm J}$ is:

$$\begin{aligned} P_{\rm J} &= \frac{1}{2}|I_{\rm in}|^2 R_{\rm J} \\ &= \frac{4R_{\rm J}R_{\rm rad}}{|Z_{\rm rad}+Z_{\rm J}|^2}\frac{|V|^2}{8R_{\rm rad}} \\ &= (1-|\Gamma_{\rm gen}|^2)P_{\rm J,max} \\ &= e_{\rm c}P_{\rm J,max}, \end{aligned} \quad (3)$$

where $P_{\rm J,max}$ is the maximum power that can be delivered to the junction when the conjugate matching condition $Z_{\rm rad} = Z_{\rm J}^*$ is satisfied or when the reflection coefficient vanishes, $\Gamma_{\rm gen} = \frac{Z_{\rm rad}-Z_{\rm J}^*}{Z_{\rm rad}+Z_{\rm J}} = 0$. The coefficient $e_{\rm c}$ is a transfer function of frequency,

$$e_{\rm c} = 1 - |\Gamma_{\rm gen}|^2 = \frac{4R_{\rm J}R_{\rm rad}}{|Z_{\rm rad}+Z_{\rm J}|^2}, \quad (4)$$

valued between 0 and 1. It estimates the fraction of power transferred to the junction, the so called absorption or coupling efficiency [6]; $e_{\rm c}$ is proportional to the real parts of the impedances $Z_{\rm J}$ and $Z_{\rm rad}$, which are derived below. Note that in the frequency response, we focus on photons with frequency f^* twice the superconducting gap near the junction, $f^* = 2\Delta/h = 105$ GHz for a 30 nm thick aluminium film, because only photons above this frequency can break Cooper pairs and directly contribute to the observed parity switching. Moreover, since the emission spectrum of a radiation source at frequencies large compared to temperature, $hf \gg k_{\rm B}T$, generally takes an exponential distribution with frequency, $e^{-hf/k_{\rm B}T}$, only photons at f^* make a significant contribution. Therefore, in our comparison of power transfer efficiency and parity-switching rate, we use $e_{\rm c}^* = e_{\rm c}(f^*)$ instead of an integrated one.

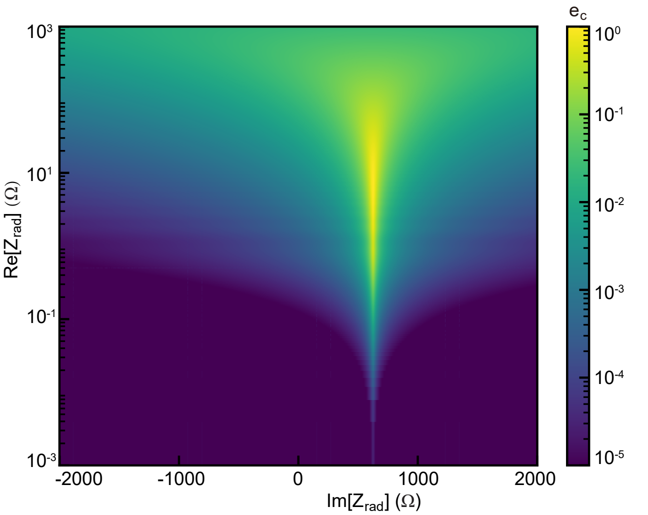

Supplementary Figure 12: Absorption efficiency versus $Z_{\rm rad}$. The brightest point is the $Z_{\rm J}^*$ value. An effective way to decrease $e_{\rm c}^*$ is to decrease $R_{\rm rad}$, while for a given $R_{\rm rad}$, $X_{\rm rad}$ has little influence on $e_{\rm c}^*$.

For radiation at a frequency above twice the superconducting gap, the junction can be seen as a resistor, with resistance equal to its normal resistance $R_{\rm n}$, shunted by the junction capacitance $C_{\rm J}$. In our design, the qubit normal resistance $R_{\rm n} \simeq 30$ kΩ, and the junction capacitance $C_{\rm J} \simeq 2.4$ fF. The junction impedance is then:

$$Z_{\rm J} = \frac{1-j\omega\tau}{1+\omega^2\tau^2}R_{\rm n}, \quad (5)$$

where $\tau = R_{\rm n}C_{\rm J}$. For a given junction impedance ($R_{\rm J} = 13$ Ω, $X_{\rm J} = -624$ Ω evaluated at f^*), we plot

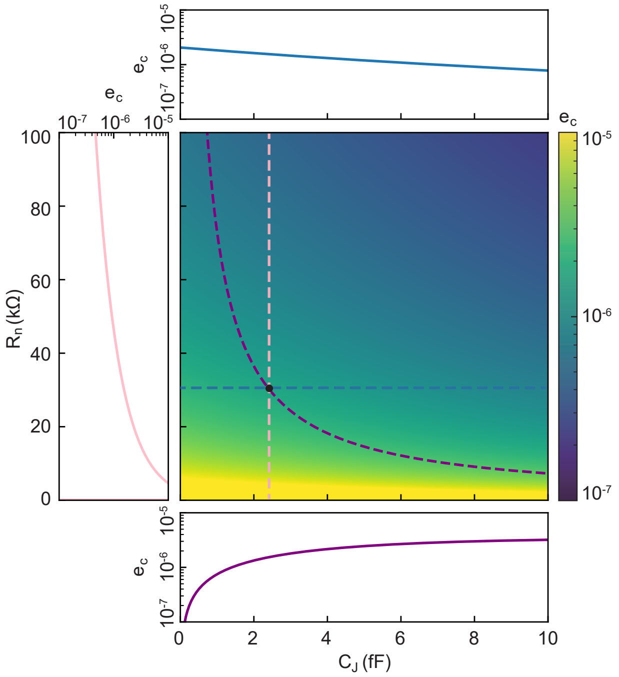

Supplementary Figure 13: Energy transfer efficiency of the qubit antenna e_{c} as a function of the junction capacitance C_{J} and the junction resistance R_{n}. Different dashed linecuts are shown in the surrounding panels in the same color coding.

in Supplementary Fig. 12 the absorption efficiency e_{c}^* as a function of the real and imaginary parts of Z_{rad} using Eq. (4). The maximum absorption occurs at $Z_{\mathrm{rad}} = Z_{\mathrm{J}}^*$, as expected from the conjugate matching condition. According to Eq. (4), reducing R_{rad} is effective in suppressing e_{c}^*. Therefore, in order to mitigate quasiparticle poisoning events, one should aim to as small as possible R_{rad} when designing the device. Besides, since the imaginary part of Z_{rad} is dominating in the denominator of Eq. (4), the more negative the imaginary part of Z_{rad}, the smaller the absorption efficiency e_{c}^*. This may be achieved by reducing the inter-pad capacitance C_0.

In Supplementary Fig. 13, we plot how the transfer efficiency e_{c} is affected by these factors according to the model described by the Eq. (4). The junction capacitance C_{J} is calculated by multiplying a specific capacitance of 75 fF/μm^2 [6] with the design value of the junction area; both junction area and specific capacitance may vary from sample to sample due to fabrication variation. It can be seen that e_{c} is only weakly dependent on C_{J} (blue dashed line and top panel); it varies less than a factor of 2 over the whole range of C_{J} (0∼10 fF). Because the junction resistance R_{n} scales inversely to the junction area, we have considered the case of covariation of R_{n} and C_{J} when the junction size fluctuates. The purple dashed line indicates where the product of R_{n} and C_{J} stays constant. In this case, e_{c} shows stronger dependence on R_{n}; this is consistent with Eq. (4) where e_{c} has a near-linear scaling with R_{J} ($\propto R_{\mathrm{n}}$) and R_{rad}. Considering typical fabrication variation, the variation in e_{c} is small around the typical working point ($R_{\mathrm{n}} = 30$ kΩ and $C_{\mathrm{J}} = 2.43$ fF) marked by the dark dot. In the paper, we deduce the value of R_{n} from the measured qubit spectrum.

B. Geometric effect on antenna impedance

To understand the impact of the key geometrical parameters to the radiation impedance of this radiating structure, we perform a parametric study. We first simulate qubit geometries with varying pad size as used in the experiment. The results are shown in Supplementary Fig. 14. It can be seen that the first two resonances shift towards lower frequencies when L increases. Since the fundamental mode is always higher than the characteristic frequency $f^* = 105$ GHz in this range, the closer these two frequencies become, the more energy the qubit absorbs. Accordingly, the real part of Z_{rad} becomes also larger, leading to higher absorption efficiency e_{c}^*, in agreement with theory. We extend the simulation with L densely sampled from 60 μm to 320 μm and with $W = 35$ μm and 60 μm. The results are shown in Fig. 3c in the main text.

We also simulate geometries with varying pad-to-ground distance d as shown in Supplementary Fig. 15. As the gap between pad and ground widen, the resonances shift towards lower frequency. In a similar argument as above, such a change leads to an increased real part of Z_{rad} and greater e_{c}^*. We extend the simulation with d densely sampled from 5 μm to 400 μm. The results are shown in Fig. 3d in the main text. The phenomenon can be understood by taking a closer look at the field distribution of the fundamental resonance mode. As shown in Supplementary Fig. 16, when d is increased from 20 μm to 400 μm, the fundamental mode is no longer confined in a narrow space right next to the pad, but expands outwards from the pad edge, leading to a longer perimeter and hence a greater effective wavelength. However, when d is large enough ($d > L$), the mode field tends to stabilize, not changing further. This qualitatively agrees with the saturation behavior when $d > 100$ μm, but we do not know the reason for the quantitative discrepancy between experimental data and simulation result.

In addition, capping the qubit helps reduce e_{c} without causing significant change to the qubit parameters such as $E_{\mathrm{J}}/E_{\mathrm{C}}$. When a square-shaped metallic cap with an edge length of 300–500 μm is placed on top of the qubit structure at a distance of 10 μm, as shown in Supplementary Fig. 17a (cf. Fig. 3a in the main text), the radiation properties of the qubit can be greatly affected. Specifically, the cap behaves as another floating ground plane located in close proximity of the paired folded slot radiator, on which currents are induced that would also contribute to the radiated field. Based on the image theory, virtual currents located on the other

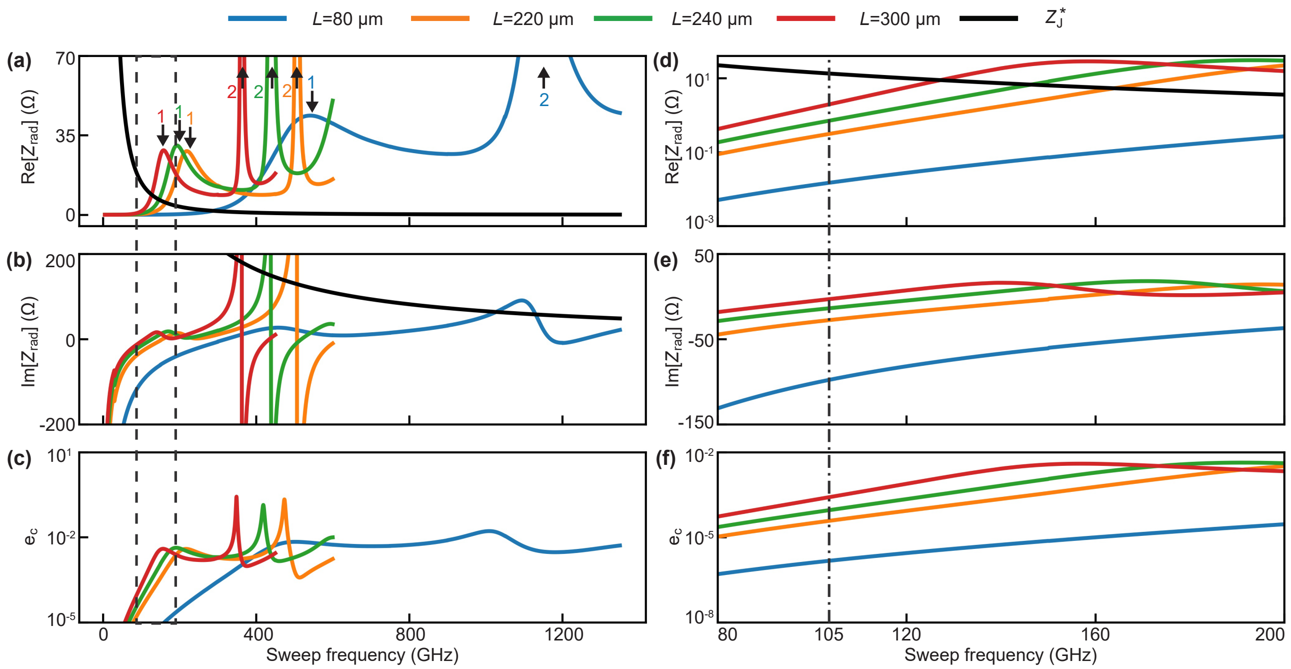

Supplementary Figure 14: Antenna impedance for different pad sizes. **a**, The real part of Z_{rad}. **b**, The imaginary part of Z_{rad}. Arrows indicate the corresponding first and second resonance modes. **c**, The absorption efficiency e_{c}. **d-f**, Zoom-in view of **a-c** between 80∼200 GHz. For the junction impedance Z_{J}, R_{n}=30 kΩ and C_{J}=2.43 fF.

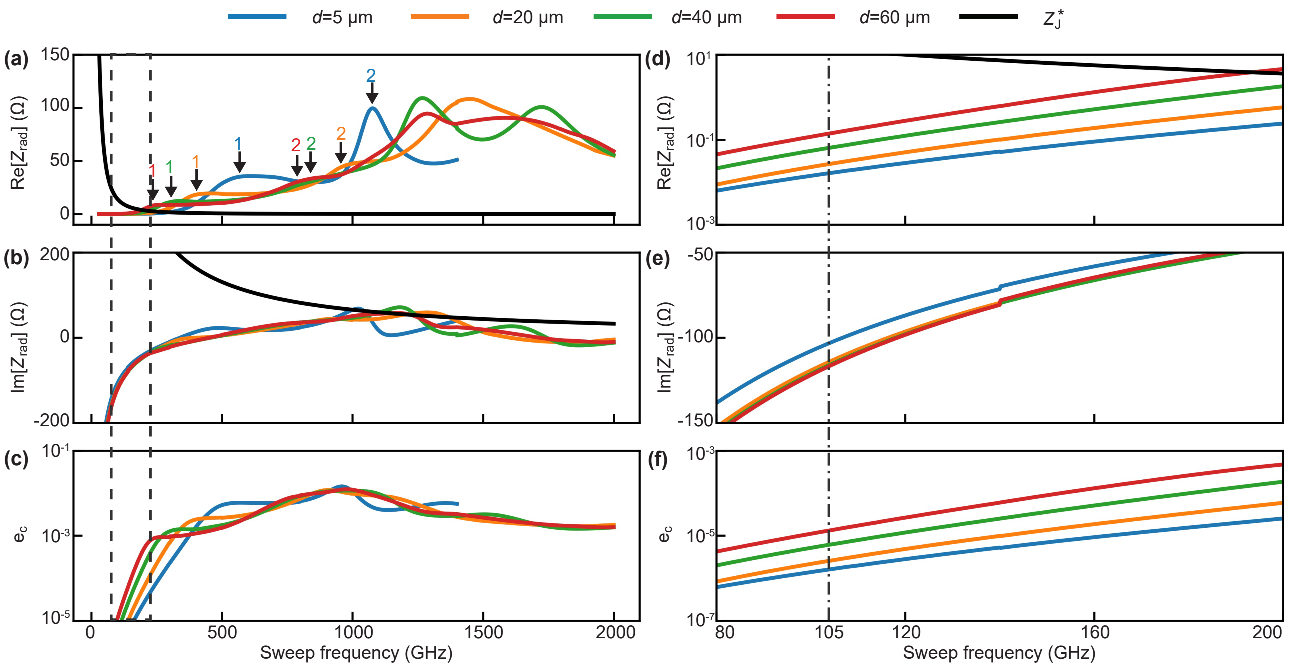

Supplementary Figure 15: Antenna impedance vs pad-to-ground distance. **a**, The real part of Z_{rad}. **b**, The imaginary part of Z_{rad}. Arrows indicate the corresponding first and second resonance modes. **c**, The absorption efficiency e_{c}. **d-f**, Zoom-in view of **a-c** between 80∼200 GHz. For the junction impedance Z_{J}, R_{n}=30kΩ and C_{J}=2.43 fF.

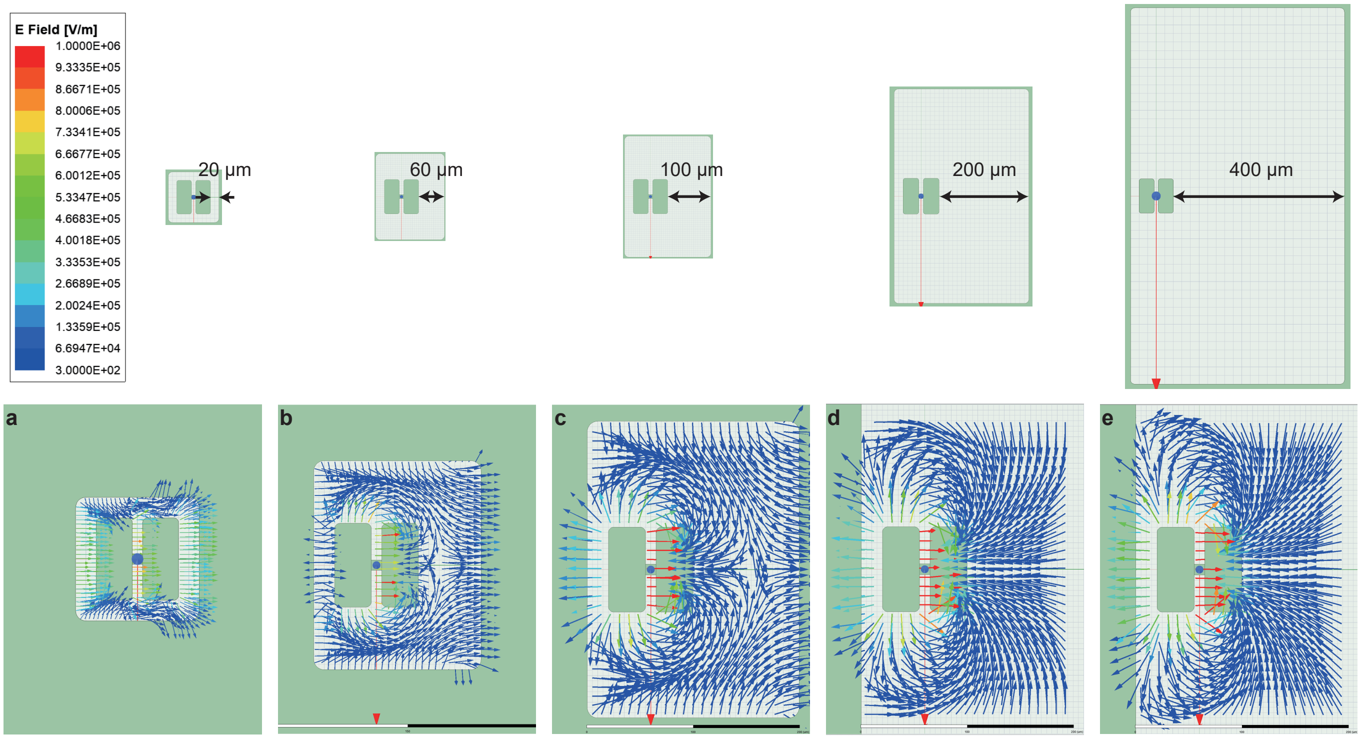

Supplementary Figure 16: E-field plot of the antenna mode for qubits with different pad-to-ground distance. a∼e, $d = 20 \sim 400$ μm. All qubits have same pad length $L = 80$ μm and pad width $W = 35$ μm. As the pad-to-ground distance d increases, the mode field spreads into the gap, and changes the effective wavelength. When d is large enough ($d > L$), the effective wavelength tends to saturate at a fixed value.

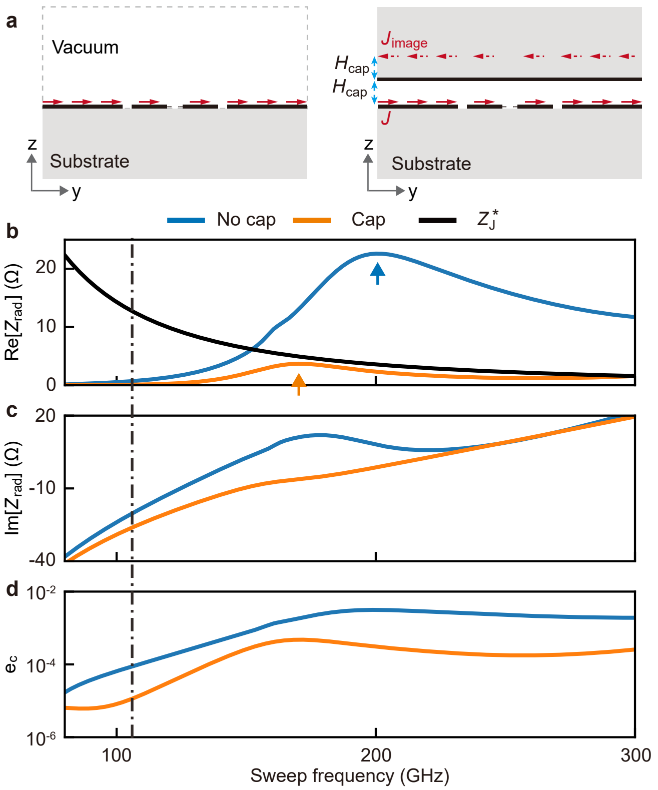

Supplementary Figure 17: Antenna impedance with and without cap. **a**, The current distribution with and without cap. The distance between chips is about 10 μm. **b**, The real part of $Z_{\rm rad}$. **c**, The imaginary part of $Z_{\rm rad}$. **d**, The absorption efficiency $e_{\rm c}$. For the junction impedance $Z_{\rm J}$, $R_{\rm n}$=30 kΩ and $C_{\rm J}$=2.43 fF.

side of the cap flowing in the horizontal directions can be used to evaluate the impact of the added floating ground. Since the virtual image currents are pointing into directions opposite to those flowing on the qubit, i.e. they are out of phase to each other, and the vertical spacing is greatly smaller than the wavelength of interest, the radiated fields from the two kinds of currents will cancel each other, thereby resulting in a significantly reduced radiation impedance [12] and $e_{\rm c}$, as shown in Supplementary Fig. 17b-d. In other words, the radiator can be considered to be shorted out by the added metallic cap at a deep-subwavelength distance. We extend the simulation with L densely sampled from 80 μm to 260 μm and with a cap sufficiently large (500×500 μm^2) to cover the qubit. The results are shown in Fig. 3c of the main text.

SUPPLEMENTARY NOTE 6: OFFSET CHARGE STABILITY

In the experiment monitoring long-term offset charge stability, we simultaneously sweep the bias of all six qubits from sample S1 and probe their readout resonators. Every scan takes 24 s, later fitted to identify zero bias. The scan is repeated for about 40 hours and divided into 3 sections. Trajectories of all qubits are shown in Supplementary Fig. 18(a-c), which records bias charge fluctuations over time.

We find that in our devices, both the frequency and the amplitude of charge jumps are considerably less than the ones in Ref. [11]. We attribute the improvement to the difference in qubit geometries: it can be understood from the perspective of the effective volumes of the charge sensitive regions. Take the example of the circular transmon from Ref. [11], which is a round-shaped pad with diameter of 140 μm. The calculated induced charge on the pad given one electron charge at a certain location in the substrate is shown in Supplementary Fig. 18d. The dashed contour line encircles the region where the induced charge is greater than 0.1 e. The total volume of this charge-sensitive region is about 1.1×10^7 μm^3. Such a volume can be reduced by simply making the qubit smaller. For example, Supplementary Fig. 18e shows the case in which the diameter of the circular transmon is reduced to 80 μm, giving a charge-sensitive volume of 1.5×10^6 μm^3. Though our floating transmon is similar in size as the small circular transmon, the charge-sensitive volume is nevertheless smaller, being only 5.0×10^5 μm^3 (Supplementary Fig. 18f). The floating design is only sensitive to the differential charge induced on the two pads, so charges beneath the gap between the pads (the $x=0$ plane) induce equal amount of charges on the pads due to symmetry and thus do not contribute to the offset bias. The effective volume can be considered similarly to a scattering cross section, which is proportional to the occurrence rate of observed events. The effective volume in our design is almost two orders of magnitude smaller than the circular transmon of Ref. [11]; this agrees with the ratio between the observed rates of charge jumps ($>0.1\,e$), 0.007-0.063 mHz in this work versus 1.35 mHz in Ref. [11].

For capped floating qubits with different pad length L but same pad-to-ground distance d, the simultaneous charge stability monitoring is shown in Supplementary Fig. 19(a-b). Supplementary Fig. 19c shows the amplitudes and total counts of all offset charge jumps ($|\Delta q|$) that are greater than $0.1\,e$ extracted from Supplementary Fig. 19(a-b). The charge jump rates are about 0.015-0.077 mHz.

To exclude the possibility of the measured charge-parity switching rate being affected by charge jumps, we also measured Γ_P at different offset charge $n_{\rm g}$ spanning more than a full period. A typical set of data is shown in Supplementary Fig. 20. We do not observe significant bias dependence in our devices; this is in agreement with theoretical expectations: at leading order the matrix element determining the parity switching rate is independent of $n_{\rm g}$ [13, 14], and while the spectral density depends on $n_{\rm g}$ through the energy difference ϵ_0 between the state of opposite parities, this dependence is

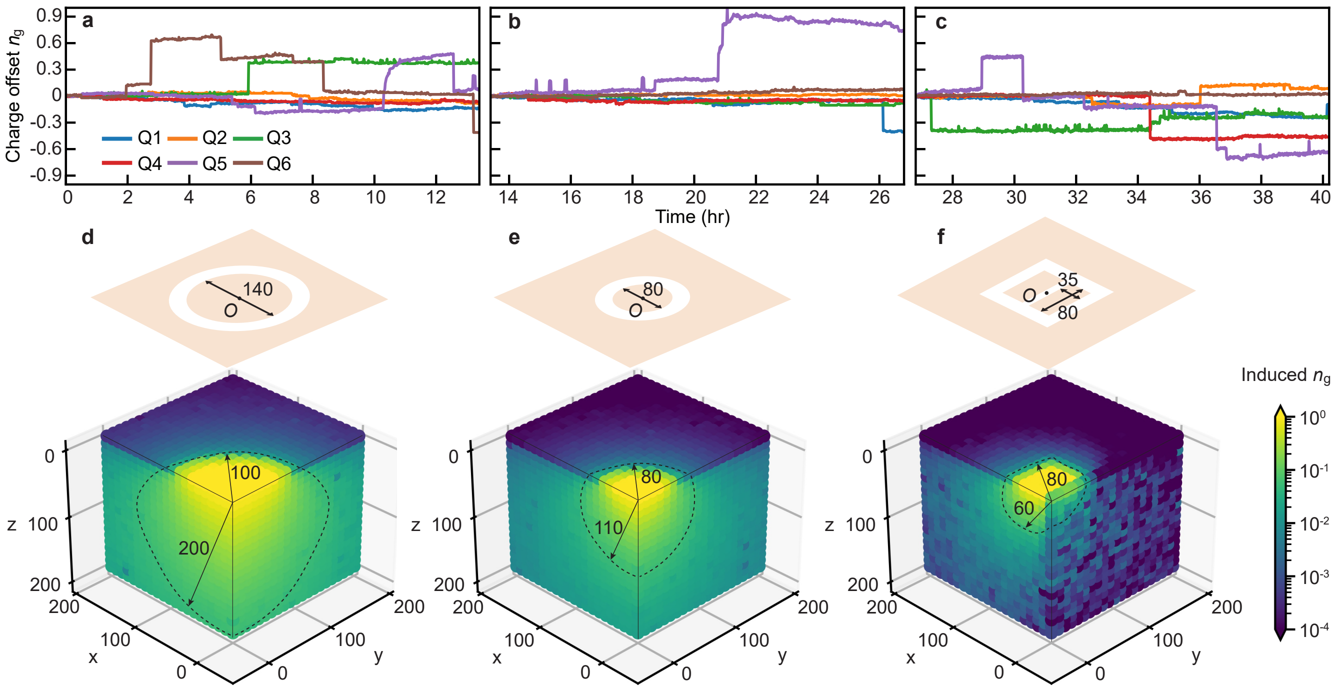

Supplementary Figure 18: Offset charge stability. a-c, Temporal trajectories of offset charge drift recorded during three separate sections over a total of 40 hours. The offset is reset at the beginning of each section. **d-f**, Calculated offset charge induced by a test point charge in the substrate with **d**, a grounded transmon with a single circular pad (diameter 140 μm, as used in Ref. [11]); **e**, a grounded transmon with a smaller circular pad (diameter 80 μm); and **f**, a floating transmon with two rectangular pads (each 80 μm×35 μm) on the top surface. O is the original point (0,0,0) in the Cartesian space in which the coordinates are the location of the test charge. We show cross-sectional planes for $x = 0$ and $y = 0$, so a quarter of the substrate is displayed. The dashed line denotes the contour of $0.1\,e$ induced charge. Note that in the floating transmon case, the induced offset charge vanishes on the $x = 0$ plane because the induced charges are symmetric on the two pads.

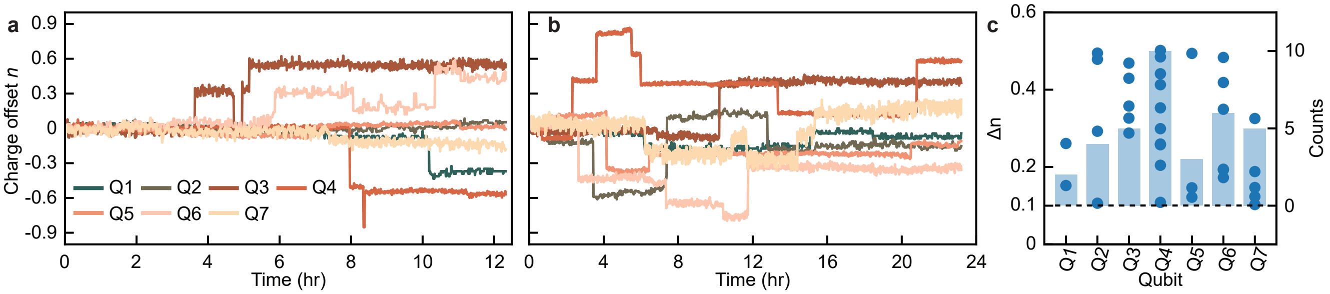

Supplementary Figure 19: Offset charge stability of capped floating qubits. a-b, Temporal trajectories of offset charge drift recorded during two separate sections over a total of 36 hours. The offset is reset at the beginning of each section. **c**, Amplitudes (left axis) and total counts (right axis) of all offset charge jumps ($|\Delta q|$) that are greater than $0.1\,e$, identified from the data in **a-b**.

small in the parameter of order $\epsilon_0/2\Delta$ [14].

SUPPLEMENTARY NOTE 7: QUBIT CALIBRATION AND CHARACTERIZATION

Due to random parity switching, to calibrate and characterize qubits of small E_J/E_C ratio requires repeated measurements. As shown in the example of qubit spectrum measurement in Supplementary Fig. 21a, the qubit spectral peaks at $n_g = 0$ of both parities become visible by plotting together repeated scans, even though the signal may switch between parities during a single scan. After finding the qubit frequency, we perform Rabi oscillations, to calibrate single-qubit gates, and then perform T_1 and T_2 measurements, as shown in Supplementary Fig. 21b-d.

We also measured the temperature dependence of coherence properties. As shown in Supplementary Fig. 22, the energy relaxation rate $\Gamma_1 = 1/T_1$ is largely temperature independent below 100 mK. This

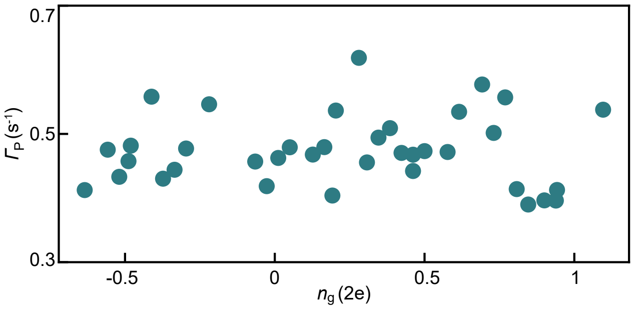

Supplementary Figure 20: Measured Γ_P at different charge offset bias.

is consistent with the fact that in our model for the temperature dependence of Γ_P (see Supplementary Note 8) up to this temperature only a small fraction (few percent) of the pads' quasiparticle density $x_{\rm qp}$ can be thermally excited, so that even in the worst case the quasiparticles would limit the relaxation time to several hundred microseconds. Meanwhile the increase of the pure-dephasing rate Γ_ϕ with temperature can be well explained by dephasing induced by thermal photons in the readout resonator according to [15]

$$\Gamma_\phi = \frac{\kappa^2}{\kappa^2+4\chi^2}\frac{4\chi^2}{\kappa}\bar{n}\,, \tag{6}$$

where $\bar{n} = 1/(e^{\hbar\omega_{\rm r}/k_{\rm B}T}-1)$ is the thermal photon number in the resonator, $\kappa = 0.8\,$MHz is the resonator decay rate, and $\chi = 0.4\,$MHz the dispersive shift due to the qubit state (not its parity).

SUPPLEMENTARY NOTE 8: TEMPERATURE DEPENDENCE OF THE PARITY SWITCHING RATE

The change of parity in the state of a superconducting qubit is a clear signature of a transition mediated by quasiparticles. Generically, the rate Γ_P of a parity-changing transition can have two contributions: one, $\Gamma_{\rm qp}$, originating from the tunnelling of a quasiparticle from one side of the junction to the other; the second, $\Gamma_{\rm pb}$, caused by the absorption of a Cooper pair-breaking photon at the junction accompanied by the generation of one quasiparticle on each side of the junction (for low quasiparticle densities, the opposite process of quasiparticle recombination with photon emission can be neglected):

$$\Gamma_P = \Gamma_{\rm qp} + \Gamma_{\rm pb}\,. \tag{7}$$

As the pair-breaking photon energy is larger than 2Δ, corresponding in Al to a temperature of a few degerees Kelvin, it is reasonable to assume that the corresponding rate is independent of the temperature T at the coldest stage of the fridge, which in our experiment range from few mK up to about 100 mK. Thus, we attribute the temperature dependence to quasiparticle tunnelling.

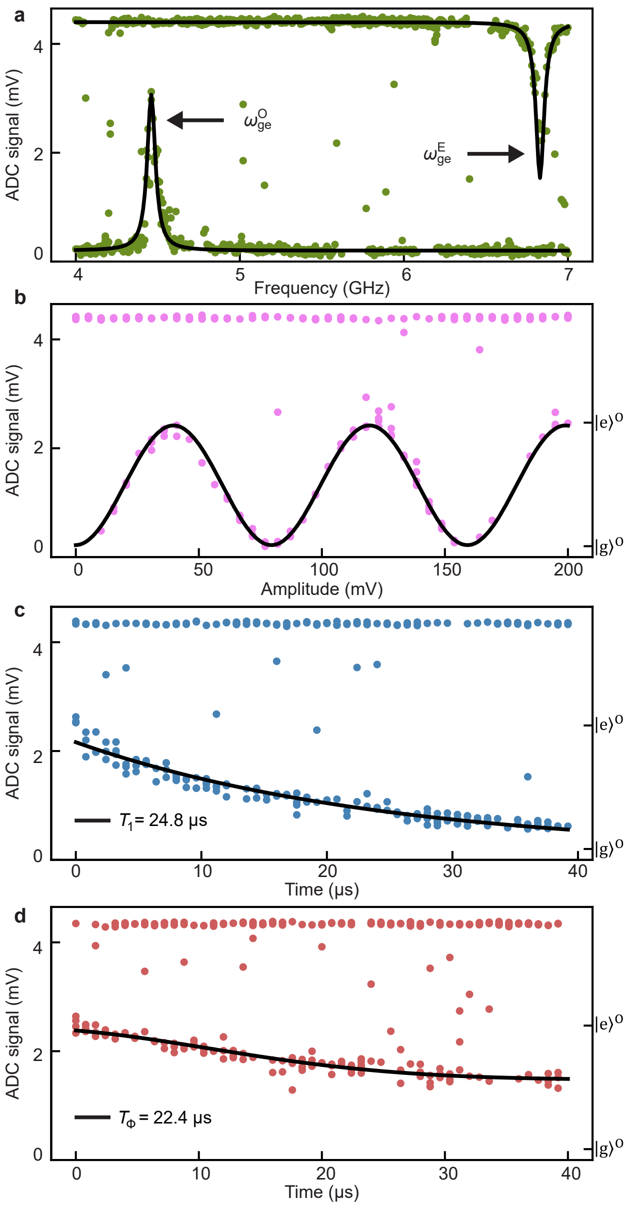

Supplementary Figure 21: Qubit characterization. **a**, Qubit spectrum measured at $n_g = 0$. Data points shown are from three repeated scans. The outliers are caused by parity switching which happens during the time taking that data point which, therefore, takes a value in between those of the two parities. **b**, Rabi oscillations obtained by driving the qubit at its odd-parity frequency $\omega_{\rm ge}^{\rm O}$. **c**, Energy relaxation measured with a π pulse calibrated from the odd-parity Rabi oscillations. Solid line is the exponential fit to $f(t) = Ae^{-t/T_1} + B$. **d**, Spin echo decay. Solid line is the fit to $G(t) = Ae^{-t/2T_1-(t/T_\phi)^2} + B$, which accounts for a Gaussian dephasing – $1/f$ noise – model and where T_1 is fixed to the energy relaxation value previously measured.

In quasiequilibrium, the temperature dependence of

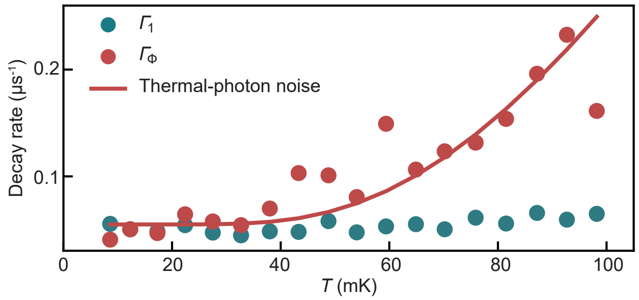

Supplementary Figure 22: Coherence versus temperature. Points: Temperature dependence of energy relaxation rate Γ_1 and pure dephasing rate Γ_ϕ measured at $n_g = 0$. Line: fit to Eq. (6) with the resonator decay rate $\kappa = 0.8$ MHz and the dispersive shift $\chi = 0.4$ MHz.

$\Gamma_{\rm qp}$ can be obtained using the results of Ref. [13]:

$$\Gamma_{\rm qp}(T) = \frac{16E_{\rm J}}{\Delta} c_0^2 \frac{\epsilon_0}{h} e^{-(\Delta-\mu)/k_{\rm B}T} F\left(\frac{\epsilon_0}{2k_{\rm B}T}, \frac{k_{\rm B}T}{2\Delta}\right), \quad (8)$$

where ϵ_0 is the energy difference between the two (ground) states of different parity, c_0 is the matrix element between those two states of the operator $\cos(\hat{\phi}/2)$, μ is an effective chemical potential that encapsulates the deviation from thermal equilibrium, and the function F is

$$F(x,y) = \cosh(x)[K_1(x) - xyK_0(x)]\,, \quad (9)$$

where K_i are the modified Bessel function of the second kind. The above formula is an approximate expression which is accurate so long as $y \lesssim 0.1$ and $xy \lesssim 0.08$. We note that $E_{\rm J}$, ϵ_0, and c_0 can be extracted from the spectroscopic data and hence, along with temperature, they are not free parameters. Not known are the values of the gap Δ at the junction and of the chemical potential, which we consider next.

As discussed in the main text, our samples consist of two pads of thickness 100 nm connected by two thinner superconducting strips (30 to 40 nm thickness), with a Josephson tunnel junction formed where the strips overlap. It is well known [16] that the gap in Al films depends on their thickness; for thick films, such as those of the pads, the gap is close to the bulk value Δ_0 =180 μeV (Δ_0/h =43.52 GHz). The gap increases as thickness decreases, and for 30 nm thick films values between 200 and 220 μeV (48.36 to 53.20 GHz) were reported in Ref. [17]. An energy difference of 20 (40) μeV corresponds to a temperature of about 230 (460) mK; therefore, at temperatures well below this value the pads act as traps for quasiparticles in the manner studied in Ref. [18]. This means that at low temperatures the quasiparticles should be largely confined to the pads and hence, in qualitative agreement with our experimental data, the parity switching rate should be independent of temperature. As temperature increases, quasiparticles can be thermally excited from the pads to the strips and hence reach the junction (cf. Fig. 1e in the main text), which could explain the increase in Γ_P with temperature.

To make the above consideration quantitative, we make the simplifying assumption that in the pads there is a fixed normalized density of quasiparticle $x_{\rm qp} \ll 1$. This density can be related to the effective chemical potential μ through the formula:

$$x_{\rm qp} \simeq \sqrt{\frac{2\pi k_{\rm B}T}{\Delta_0}} e^{-(\Delta_0-\mu)/k_{\rm B}T} \quad (10)$$

We can invert this expression to obtain the chemical potential as function of T, Δ_0, and $x_{\rm qp}$. Substituting the result into Eq. (8) we can finally write:

$$\Gamma_P(T) = \Gamma_P(0) + \frac{16E_{\rm J}}{\Delta} c_0^2 \frac{\epsilon_0}{h} e^{\frac{-(\Delta-\Delta_0)}{k_{\rm B}T}} x_{\rm qp} \sqrt{\frac{\Delta_0}{2\pi k_{\rm B}T}} F\left(\frac{\epsilon_0}{2k_{\rm B}T}, \frac{k_{\rm B}T}{2\Delta}\right) \quad (11)$$

where $\Gamma_P(0)$ accounts for all possible temperature-independent contribution to Γ_P (including $\Gamma_{\rm pb}$, but also other possible sources of parity switching). Thus, we have three free parameters: $\Gamma_P(0)$, Δ (the gap at the junctions), and $x_{\rm qp}$, that can be used in comparing theory to experiment. Note that of these parameters, only Δ and $x_{\rm qp}$ appear in the temperature-dependent term of Eq. (11), while $\Gamma_P(0)$ is independent of temperature.

We perform such a comparison for fifteen qubits belonging to four samples, Sample S1, S4, S5, and S8; the corresponding parameters are given in Supplementary Table II. For the first three samples, the experimental data with fit (in log scale) are presented in Fig. 4 of the main text. When fitting the data, we require Δ to be the same for qubits of a given sample, since the thicknesses of their superconducting arms are expected to be the same; this leaves only $x_{\rm qp}$ as a parameter to fit the temperature dependence for a given qubit in a chip. The normalized quasiparticle densities are of order 10^{-7}, comparable to those reported in the literature [19, 20]. It should be noted that even at 100 mK the expected thermal equilibrium value for $x_{\rm qp}$ is about 4.6×10^{-10} and hence always two orders of magnitude smaller than our extracted densities. This justifies assuming the temperature independence of the quasiparticle density in the pads, its value being determined by some non-equilibrium process. In fact, the data in Fig. 4b of the main text suggest that pair breaking at the junction contributes significantly to the non-equilibrium density $x_{\rm qp}$. In the steady-state, $x_{\rm qp}$ is determined by the balance between generation with rate g, recombination with rate r (as mentioned in the main text), and possibly single-quasiparticle trapping with rate s [20],

$$0 = g - sx_{\rm qp} - rx_{\rm qp}^2\,. \quad (12)$$

The recombination rate is material and geometry dependent; for thin films of aluminium it is estimated to be $r \approx 1/(120\,{\rm ns})$. The trapping rate s can be

	Pad length L (μm)	Pad width W (μm)	Distance d (μm)	$\omega_{\rm ge}^{\rm max}/2\pi$	$\omega_{\rm ge}^{\rm min}/2\pi$	$E_{\rm J}/h$	$E_{\rm C}/h$	ϵ_0/h	c_0^2	$\Gamma_P(0)$	Δ/h	$10^7 x_{\rm qp}$
S1−Q1	80	35	5	6.833	4.473	4.67	1.40	0.238	0.775	0.48	50.9	0.22
S1−Q2	80	35	10	6.954	4.135	4.27	1.48	0.319	0.755	0.77	50.9	1.94
S1−Q4	80	35	20	7.268	4.469	4.63	1.53	0.305	0.762	0.92	50.9	2.28
S4−Q1	80	35	5	7.845	6.754	7.61	1.30	0.0677	0.838	0.57	52.53	0.73
S4−Q2	220	35	5	5.774	5.505	6.92	0.78	0.0117	0.872	13.93	52.53	3.51
S4−Q3	240	60	5	6.068	6.067	12.25	0.44	2.18×10^{-5}	0.931	34.06	52.53	5.16
S5−Q1	80	35	5	7.246	5.740	6.25	1.31	0.112	0.817	1.59	49.50	0.35
S5−Q2	220	35	5	4.907	4.400	5.12	0.76	0.027	0.850	19.11	49.50	3.90
S5−Q3	240	60	5	6.2013	6.2012	14.66	0.37	1.6×10^{-6}	0.942	44.12	49.50	2.87
S8−Q2	80	35	5	5.566	2.281	2.35	1.29	0.490	0.691	0.42	48.55	0.96
S8−Q3	120	35	5	4.260	2.230	2.23	0.95	0.272	0.726	0.63	48.55	1.15
S8−Q5	180	35	5	4.254	3.858	4.45	0.66	0.024	0.846	1.10	48.55	0.42
S8−Q6	210	35	5	4.104	3.899	4.71	0.59	0.013	0.858	1.60	48.55	0.28
S8−Q7	260	35	5	3.832	3.712	4.84	0.49	0.005	0.871	1.60	48.55	0.70
S8−Q8	260	35	5	4.116	4.059	5.79	0.46	0.002	0.881	1.60	48.55	0.44

Supplementary Table 2: Qubit parameters. Qubit design parameters L, W, d, maximum and minimum frequencies $\omega_{\rm ge}^{\rm max/min}$, and Josephson and charging energies $E_{\rm J}$ and $E_{\rm C}$ are as in Supplementary Table I and are reported here for convenience. The energy difference between g states of different parities ϵ_0 and the squared matrix element c_0^2 are calculated numerically from $E_{\rm C}$ and $E_{\rm J}$ (although c_0^2 can be estimated using the analytical formula given in [13] within 2% percent in the worst case of S8-Q2, which has the smallest ratio E_J/E_C), while $\Gamma_P(0)$, Δ, and $x_{\rm qp}$ are found by fitting Γ_P versus temperature using Eq. (11). Numbers c_0^2 and $x_{\rm qp}$ are dimensionless, $\Gamma_P(0)$ is given in Hz, and the remaining quantities in GHz.

written as the sum of a background rate s_0 plus a term due to vortices s_v: $s = s_0 + s_v$; assuming no vortices, we take $s = s_0 \approx 0.05\,{\rm ms}^{-1} = 50\,{\rm s}^{-1}$. (For both recombination and trapping rates, their values are taken from Ref. [20].) Finally, the generation rate can in principle have several contributions, such as pair-breaking phonons being generated in the substrate by radiation [21] or pair-breaking photons absorbed directly in the pads. For our purposes, we assume that the quasiparticles generated at the junction with rate $\Gamma_P(0)$ quickly diffuse to the pads, relax by phonon emission to an energy lower than Δ (the gap in the junction's arms), and are hence trapped in the pads; in this scenario, the generation rate is

$$g = \frac{\Gamma_P(0)}{2\nu_0\Delta_0 V}\,, \tag{13}$$

where $\nu_0 = 0.73\times10^{47}\,({\rm J\ m^3})^{-1}$ is the single-spin density of states at the Fermi energy in Al, Δ_0 is the gap in the pads, and V the volume of one pad.

For the densities of interest ($x_{\rm qp} < 6\times10^{-7}$), the recombination term is smaller than the trapping one, $rx_{\rm qp} < s$, so trapping should be dominant, giving $x_{\rm qp} \sim g/s$; however, even for the largest value of $g \sim 10^{-8}\,{\rm s}^{-1}$ (corresponding to the largest $\Gamma_P(0)$ measured in the qubit with the biggest pads), the expected density would be of order $x_{\rm qp} \sim 10^{-10}$, much smaller than the measured densities. In the devices of Ref. [20], the pads consisted of a bilayer where two aluminium films were separated by a thin insulating layer of aluminium oxide; this oxide, absent in our devices, could be the source of background trapping. If trapping can be neglected, one instead finds $x_{\rm qp} = \sqrt{g/r} \sim 3\times10^{-8}$, which is still one order of magnitude smaller than the largest measured density.

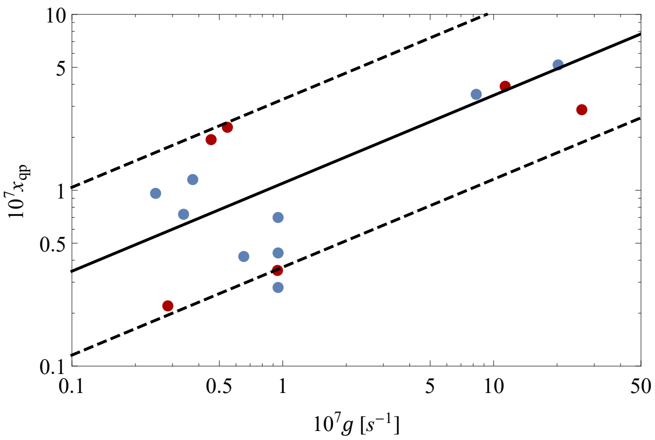

Supplementary Figure 23: Points: experimental data, where the generation rate g is calculated using Eq. (13) with $V = 4\,\mu{\rm m}^3$ the volume of a bandage; red: data from qubits in samples S1 and S5 (same as in Fig. 4b of the main text), blue: data from qubits in S4 and S8. Solid line: theory estimate using the same recombination rate r as in Ref. [20]. Dashed lines: theory estimates multiplied or divided by 3.

A possible resolution of the discrepancy is as follows: while we have assumed that the quasiparticles are trapped in the pads, they could be trapped in the bandages instead. The bandages are twice as thick as the pads (200 vs 100 nm), so their gap is likely a few μeV smaller, equivalent to a temperature of a few tens

of mK, hence lower than base temperature. Moreover, due to their small lateral dimensions ($10 \times 2\,\mu\text{m}^2$), the bandages are unlikely to host vortices, explaining the absence of trapping. For the purpose of fitting the temperature dependence of the parity switching rate, Fig. 4a of the main text, it doesn't matter if the non-equilibrium quasiparticles are activated from the pads or the bandages; however, if the bandages' volume is used in Eq. (13) to calculate the generation rate g, we find that the data is reasonably well explained (within a factor of ~ 3) by the relation $x_{\text{qp}} = \sqrt{g/r}$ with the same recombination rate r of Ref. [20], see Supplementary Fig. 23. Interestingly, this result suggests that either no significant quasiparticle generation takes place in the pads, or that the quasiparticles generated there are quickly trapped, for instance due to one or a few vortices, so that they do not reach the bandages (for reference, the trapping rate s_v by a single vortex in the largest pad is $s_v \approx 400\,\text{s}^{-1}$ and s_v is larger for smaller pads [20]).

[1] X. Y. Jin, A. Kamal, A. P. Sears, T. Gudmundsen, D. Hover, J. Miloshi, R. Slattery, F. Yan, J. Yoder, and T. P. Orlando, Physical Review Letters **114**, 565 (2015).

[2] K. Serniak, S. Diamond, M. Hays, V. Fatemi, S. Shankar, L. Frunzio, R. J. Schoelkopf, and M. H. Devoret, Phys. Rev. Applied **12**, 014052 (2019).

[3] R. Gordon, C. Murray, C. Kurter, M. Sandberg, S. Hall, K. Balakrishnan, R. Shelby, B. Wacaser, A. Stabile, J. Sleight, M. Brink, M. Rothwell, K. Rodbell, O. Dial, and M. Steffen, Appl. Phys. Lett. **120**, 074002 (2022).

[4] A. Blais, R.-S. Huang, A. Wallraff, S. M. Girvin, and R. J. Schoelkopf, Phys. Rev. A **69**, 062320 (2004).

[5] D. Ristè, C. C. Bultink, M. J. Tiggelman, R. N. Schouten, K. W. Lehnert, and L. Dicarlo, Nat. Commun. **4**, 1913 (2013).

[6] O. Rafferty, S. Patel, C. H. Liu, S. Abdullah, C. D. Wilen, D. C. Harrison, and R. McDermott, (2021), arXiv:2103.06803.

[7] J. D. Kraus, *Antennas for All Applications* (McGraw-Hill Science, 2001).

[8] D. M. Pozar, *Microwave Engineering* (John Wiley, 2011).

[9] AnsysHFSS, `https://www.ansys.com/products/electronics/ansys-hfss`.

[10] S. J. Orfanidis, *Electromagnetic Waves and Antennas* (http://eceweb1.rutgers.edu/orfanidi/ewa/, 2008) pp. 749–751.

[11] C. D. Wilen, S. Abdullah, N. A. Kurinsky, C. Stanford, L. Cardani, G. D'Imperio, C. Tomei, L. Faoro, L. B. Ioffe, C. H. Liu, A. Opremcak, B. G. Christensen, J. L. DuBois, and R. McDermott, Nature **594**, 369 (2021), 2012.06029.

[12] C. A. Balanis, *Advanced Engineering Electromagnetics* (John Wiley, 2012).

[13] G. Catelani, Phys. Rev. B **89**, 094522 (2014).

[14] M. Houzet, K. Serniak, G. Catelani, M. Devoret, and L. I. Glazman, Phys. Rev. Lett. **123**, 107704 (2019).

[15] F. Yan, S. Gustavsson, A. Kamal, J. Birenbaum, A. P. Sears, D. Hover, T. J. Gudmundsen, D. Rosenberg, G. Samach, S. Weber, J. L. Yoder, T. P. Orlando, J. Clarke, A. J. Kerman, and W. D. Oliver, Nat. Commun. **7**, 12964 (2016).

[16] P. N. Chubov, V. V. Eremenko, and Y. A. Pilipenko, Sov. Phys. JETP **28**, 389 (1969).

[17] N. A. Court, A. J. Ferguson, and R. G. Clark, Supercond. Sci. Technol. **21**, 015013 (2007).

[18] R.-P. Riwar and G. Catelani, Phys. Rev. B **100**, 144514 (2019).

[19] I. M. Pop, K. Geerlings, G. Catelani, R. J. Schoelkopf, L. I. Glazman, and M. H. Devoret, Nature **508**, 369 (2014).

[20] C. Wang, Y. Y. Gao, I. M. Pop, U. Vool, C. Axline, T. Brecht, R. W. Heeres, L. Frunzio, M. H. Devoret, G. Catelani, L. I. Glazman, and R. J. Schoelkopf, Nat. Commun. **5**, 5836 (2014).

[21] L. Cardani, F. Valenti, N. Casali, G. Catelani, T. Charpentier, M. Clemenza, I. Colantoni, A. Cruciani, G. D'Imperio, L. Gironi, L. Grünhaupt, D. Gusenkova, F. Henriques, M. Lagoin, M. Martinez, G. Pettinari, C. Rusconi, O. Sander, C. Tomei, A. V. Ustinov, M. Weber, W. Wernsdorfer, M. Vignati, S. Pirro, and I. M. Pop, Nat. Commun. **12**, 2733 (2021).
